# Biallelic *HEPHL1* variants impair ferroxidase activity and cause an abnormal hair phenotype

**DOI:** 10.1371/journal.pgen.1008143

**Published:** 2019-05-24

**Authors:** Prashant Sharma, Marie Reichert, Yan Lu, Thomas C. Markello, David R. Adams, Peter J. Steinbach, Brie K. Fuqua, Xenia Parisi, Stephen G. Kaler, Christopher D. Vulpe, Gregory J. Anderson, William A. Gahl, May Christine V. Malicdan

**Affiliations:** 1 NIH Undiagnosed Diseases Program, Common Fund, National Human Genome Research Institute, National Institutes of Health, Bethesda, Maryland, United States of America; 2 Office of the Clinical Director, National Human Genome Research Institute, National Institutes of Health, Bethesda, Maryland, United States of America; 3 Iron Metabolism Laboratory, QIMR Berghofer Medical Research Institute, Brisbane, Queensland, Australia; 4 Medical Genetics Branch, National Human Genome Research Institute, National Institutes of Health, Bethesda, Maryland Bethesda, Maryland, United States of America; 5 Center for Molecular Modeling, Center for Information Technology, National Institutes of Health, Bethesda, Maryland, United States of America; 6 Department of Medicine, University of California, Los Angeles, United States of America; 7 Section on Translational Neuroscience, Molecular Medicine Branch, *Eunice Kennedy Shriver* National Institute of Child Health and Human Development, National Institutes of Health, Bethesda, Maryland, United States of America; 8 Center for Environmental and Human Toxicology, Department of Physiological Sciences, University of Florida, Gainesville, Florida, United States of America; Stanford University School of Medicine, UNITED STATES

## Abstract

Maintenance of the correct redox status of iron is functionally important for critical biological processes. Multicopper ferroxidases play an important role in oxidizing ferrous iron, released from the cells, into ferric iron, which is subsequently distributed by transferrin. Two well-characterized ferroxidases, ceruloplasmin (CP) and hephaestin (HEPH) facilitate this reaction in different tissues. Recently, a novel ferroxidase, Hephaestin like 1 (HEPHL1), also known as zyklopen, was identified. Here we report a child with compound heterozygous mutations in *HEPHL1* (NM_001098672) who presented with abnormal hair (pili torti and trichorrhexis nodosa) and cognitive dysfunction. The maternal missense mutation affected mRNA splicing, leading to skipping of exon 5 and causing an in-frame deletion of 85 amino acids (c.809_1063del; p.Leu271_ala355del). The paternal mutation (c.3176T>C; p.Met1059Thr) changed a highly conserved methionine that is part of a typical type I copper binding site in HEPHL1. We demonstrated that HEPHL1 has ferroxidase activity and that the patient’s two mutations exhibited loss of this ferroxidase activity. Consistent with these findings, the patient’s fibroblasts accumulated intracellular iron and exhibited reduced activity of the copper-dependent enzyme, lysyl oxidase. These results suggest that the patient’s biallelic variants are loss-of-function mutations. Hence, we generated a *Hephl1* knockout mouse model that was viable and had curly whiskers, consistent with the hair phenotype in our patient. These results enhance our understanding of the function of HEPHL1 and implicate altered ferroxidase activity in hair growth and hair disorders.

## Author summary

Multi-copper ferroxidases play a critical role in maintaining iron homeostasis in humans. Two well-characterized ferroxidases, ceruloplasmin and hephaestin, facilitate iron transport in different tissues by oxidizing ferrous iron to the ferric form, which is subsequently carried by transferrin. Hephaestin like 1 (HEPHL1) is a new member of the multicopper oxidase family, with a typical six-domain multi-copper ferroxidase structure containing type I, type II and binuclear type III copper binding sites, predicted to coordinate six copper atoms. The physiological role of HEPHL1 in iron homeostasis is unknown. In this study, we provide initial insights into a possible physiological role for HEPHL1 by functionally characterizing two different mutations found in a patient who presented with abnormal hair (pili torti and trichorrhexis nodosa), combined-type ADHD, speech articulation disorder, increased joint mobility, severe heat intolerance, and chronic leg pain. Whole exome sequencing revealed biallelic loss of function mutations in *HEPHL1*.We show that both mutations adversely affect the ferroxidase activity of HEPHL1. Remarkably, complete ablation of *Hephl1* in the mouse leads to a curly whisker (vibrissae) hair phenotype, supporting an important role for the ferroxidase activity of HEPHL1 in hair growth and hair disorders.

## Introduction

Iron is an essential trace element and constituent of important cellular proteins that include hemoglobin, myoglobin, flavoproteins, cytochromes and various non-heme enzymes. Transfer of electrons via oxidation–reduction (redox) reactions results in the conversion of iron between its ferrous [Fe (II)] and ferric [Fe (III)] forms. This property allows iron to participate in vital biological processes including oxygen transport, DNA biosynthesis and oxidative phosphorylation [1]. In mammals, iron homeostasis is precisely regulated to ensure proper iron acquisition, transfer, and storage while also preventing the donation of electrons to molecular oxygen that would otherwise lead to the generation of toxic free radicals [2].

Dietary iron is absorbed predominantly in the duodenum and traverses both the apical and basolateral membranes of absorptive epithelial cells to reach the plasma. For non-heme iron, one or more ferrireductases (e.g., DCTYB) on the apical surface of duodenal cells first converts Fe (III) to Fe (II) [3]. Fe (II) is then imported by an apical divalent metal-ion transporter (DMT1) [4–6] into the cytosol where it can be stored in the iron-storage molecule ferritin or exported into plasma by the basolateral iron exporter ferroportin [7, 8]. The transmembrane protein ferroportin transports Fe (II) into the plasma, but Fe (II) must be oxidized to Fe (III) for incorporation into transferrin. This function is carried out by hephaestin (HEPH), which is a member of the multicopper oxidase family that facilitates the conversion of Fe (II) to Fe (III) in an enzymatic reaction that uses oxygen as an electron acceptor [9]. HEPH is the predominant multicopper ferroxidase expressed in the basolateral membrane of absorptive intestinal cells [10]. Mice harboring loss-of-function mutations in *Heph* (Sex-linked anemia or Sla mice) show marked accumulation of iron in the intestinal mucosa and systemic iron deficiency, owing to a deficit in iron export [11]. Ablation of *Heph* either specifically in the intestine or in the whole body also leads to iron accumulation in duodenal enterocytes and reduction in intestinal iron absorption [12]. These findings show the specific role of *Heph* in intestinal enterocytes in maintaining whole body iron homeostasis.

Ceruloplasmin (CP), a paralog of HEPH and the principal ferroxidase in plasma, oxidizes Fe (II) to Fe (III) and is involved in the release of Fe (III) from multiple cell types, allowing iron to bind transferrin in blood and extracellular fluid [9, 13]. Genetic deficiency of CP (aceruloplasminemia) in humans leads to iron overload in the liver, brain and pancreas, and results in progressive neurological disease and diabetes [14]. Similarly, mutations in the *Cp* gene in mice lead to decreased iron export and increased iron retention in the liver and spleen [13].

HEPHL1 or zyklopen, another member of the multicopper oxidase family, was identified and studied in BeWo cells, a placental cell line. Molecular modeling of HEPHL1 revealed a typical six-domain multi-copper ferroxidase structure with type I, type II and binuclear type III copper binding sites, predicted to coordinate six copper atoms[15]. Like HEPH, HEPHL1 is predicted to anchor to the plasma membrane due to the presence of a putative transmembrane region at the C-terminus. A role for HEPHL1 in mediating iron efflux in placental trophoblasts during iron transport from mother to developing fetus has been proposed [15–17], but not yet confirmed; the role of HEPHL1 in iron transport remains uncertain.

In this study, we demonstrate a potential physiological role for HEPHL1 based upon two different pathogenic *HEPHL1* mutations found in a patient who clinically presented with abnormal hair (pili torti and trichorrhexis nodosa), combined-type attention deficit hyperactivity disorder (ADHD), speech articulation disorder, increased joint mobility, severe heat intolerance, and chronic leg pain. We show that each mutation adversely affects the ferroxidase activity and post-translational modification of HEPHL1. Remarkably, complete ablation of *Hephl1* in mouse leads to a curly whisker (vibrissae) hair phenotype, supporting an important role for the ferroxidase activity of HEPHL1 in hair development.

## Results

### Clinical and laboratory findings in a boy with abnormal hair

**Fig 1:**
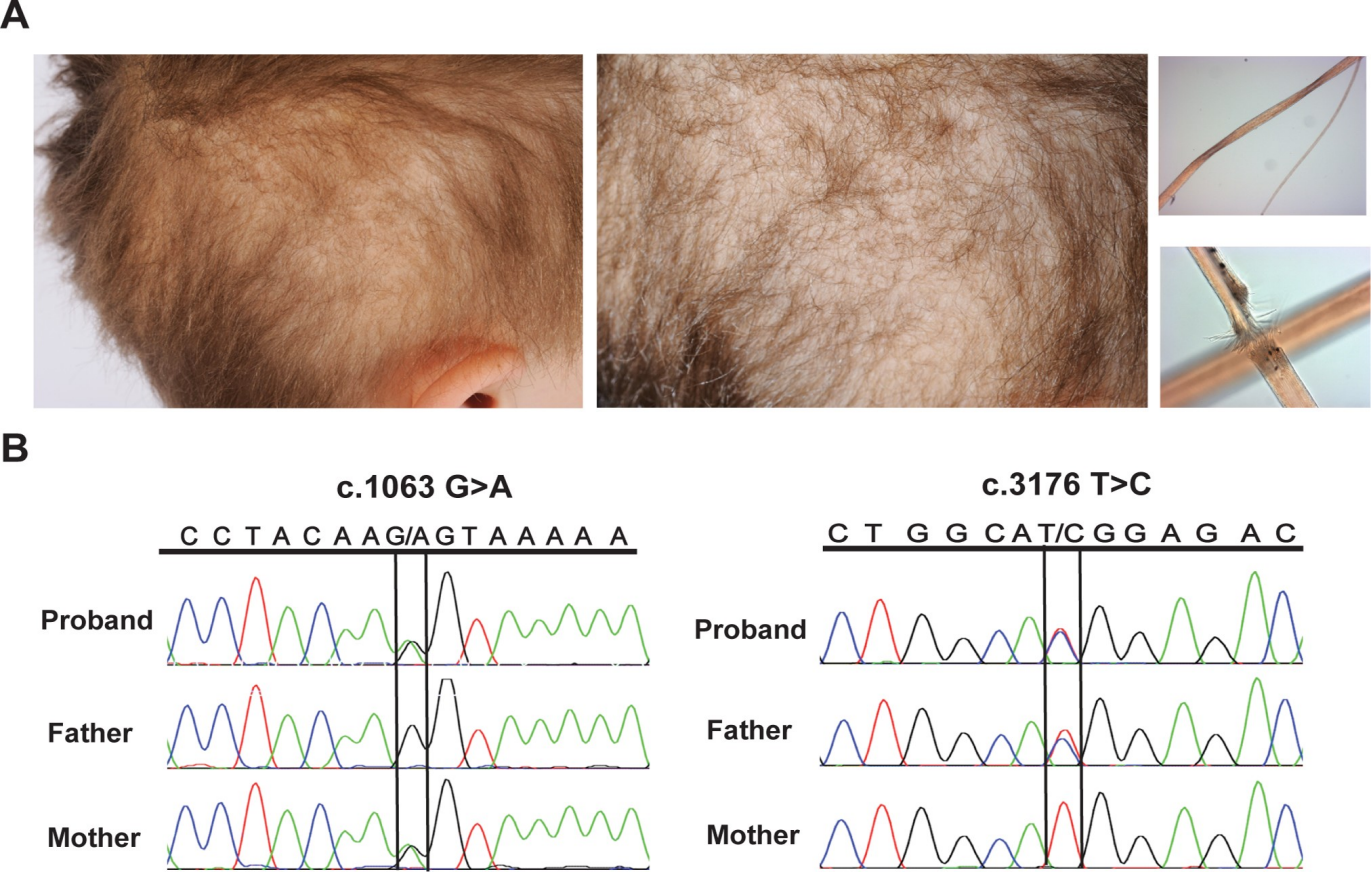
Hair phenotype and identification of *HEPHL1* mutations. (A) Patient photographs taken at age 5 years show hair abnormalities. Sparse distribution of hair in the temporal area (left), and coarse hair texture (middle) are shown. Light microscopic examination of hairs showing pili torti (right upper) and trichorrhexis nodosa (right lower). (B) Sanger sequencing chromatograms showing biallelic mutations in the proband. The proband is a compound heterozygous for NM_001098672 (HEPHL1): c.1063G>A; p. Ala355Thr (inherited from the mother, left) and NM_001098672 (HEPHL1): c.3176T>C; p. Met1059Thr (inherited from the father, right).

We evaluated a 5-year old Caucasian male of non-consanguineous Native American and Mexican descent with abnormal hair growth, and early cognitive delays (see S1 Text for a full clinical phenotype description). At birth, his hair was thick and black and distributed evenly on his scalp. He had no eyebrows but did have full eyelashes. Anterior hair loss gradually progressed to total alopecia by six months of age. His hair then regrew in a patchy distribution, sparsely in the temporal areas and more along the crown. Physical examination at age 5 years revealed elfin facies, absent lateral third of his eyelashes, sparse eyebrows and coarse hair texture (Fig 1A, left), reminiscent of X-linked recessive Menkes disease (MIM 309400). Light microscopy of both short and long hair demonstrated pili torti and trichorrhexis nodosa (Fig 1A, right).

Given the possible diagnosis of Menkes disease, we performed relevant biochemical testing. The patient had normal levels of serum copper (115 μg/dL; normal range 85–150) and ceruloplasmin (34.7 mg/dL; normal range 24–46), and normal plasma catecholamines (norepinephrine 95 pg/mL; normal range 80–498, epinephrine estimation <23 pg/mL with an interfering peak present, and dopamine 17 pg/mL; normal range 3–46). Molecular analysis of the Menkes disease-associated gene, *ATP7A*, was performed using multiplex PCR and direct DNA sequencing as previously described [18]. The patient had a previously reported single nucleotide polymorphism, H1178Y, in exon 18 of *ATP7A*; this polymorphism, however, occurs in the normal population at an expected frequency of 1% [19]. No other sequence alterations in the coding regions or intronic splice junctions of the *ATP7A* gene were found. Together, the biochemical and genetic analyses argued against the diagnosis of Menkes disease. A possible diagnosis of Ectrodactyly-ED-Clefting (EEC), Rapp Hodgkin ((MIM 129400) or a related syndrome was also ruled out by our failure to find any disease-associated mutation in PCR amplified genomic DNA for analysis of *TP63* (p63; TP73L) exons 5–8 and 13–14, and their flanking splice sites.

### Exome sequencing reveals biallelic mutations in *HEPHL1*

Subsequent whole exome sequencing (WES) and follow-up Sanger sequencing of genomic DNA from the proband identified compound heterozygous mutations in the *HEPHL1* gene; both parents were heterozygous carriers (Fig 1B). The maternal variant (NM_001098672: c.1063G>A; p. Ala355Thr) is predicted to cause a canonical splice site interruption while the paternal variant (NM_001098672: c.3176T>C; p. Met1059Thr) is a missense mutation leading to a Met1059Thr change in a copper oxidase domain of HEPHL1. Both variants are present in the Exome Aggregation Consortium (ExAC) Browser and Genome Aggregation Database (gnomAD) (see URLs) in the European population at extremely low frequencies (0.0001074 and 0.000101 for the maternal and paternal variants, respectively). Neither variant is present in the homozygous state in the ExAC/gnomAD databases. Search for other patients with biallelic variants in *HEPHL1* using GeneMatcher and through other collaborators did not yield any matches. The combined annotation dependent depletion (CADD) Phred scores, which rank the deleteriousness of single nucleotide variants within the human genome, were 26.1 and 27.3 for the maternal and paternal variants, respectively. Both variants are predicted to be disease causing by Mutation Taster (see URLs) and "probably damaging" by PolyPhen-2 (see URLs). Webserver wInterVar (see URLs), which classifies genetic variants according to the ACMG/AMP 2015 guidelines [20], ranked both variants as pathogenic with evidence codes PS3, PM4, PP3 and PS3, PM1, PP3 for the p. Ala355Thr and p. Met1059Thr variants, respectively. These *in silico* analysis tools support the contention that both variants are likely to affect the expression and function of HEPHL1.

### Molecular analysis of *HEPHL1* mutations

**Fig 2:**
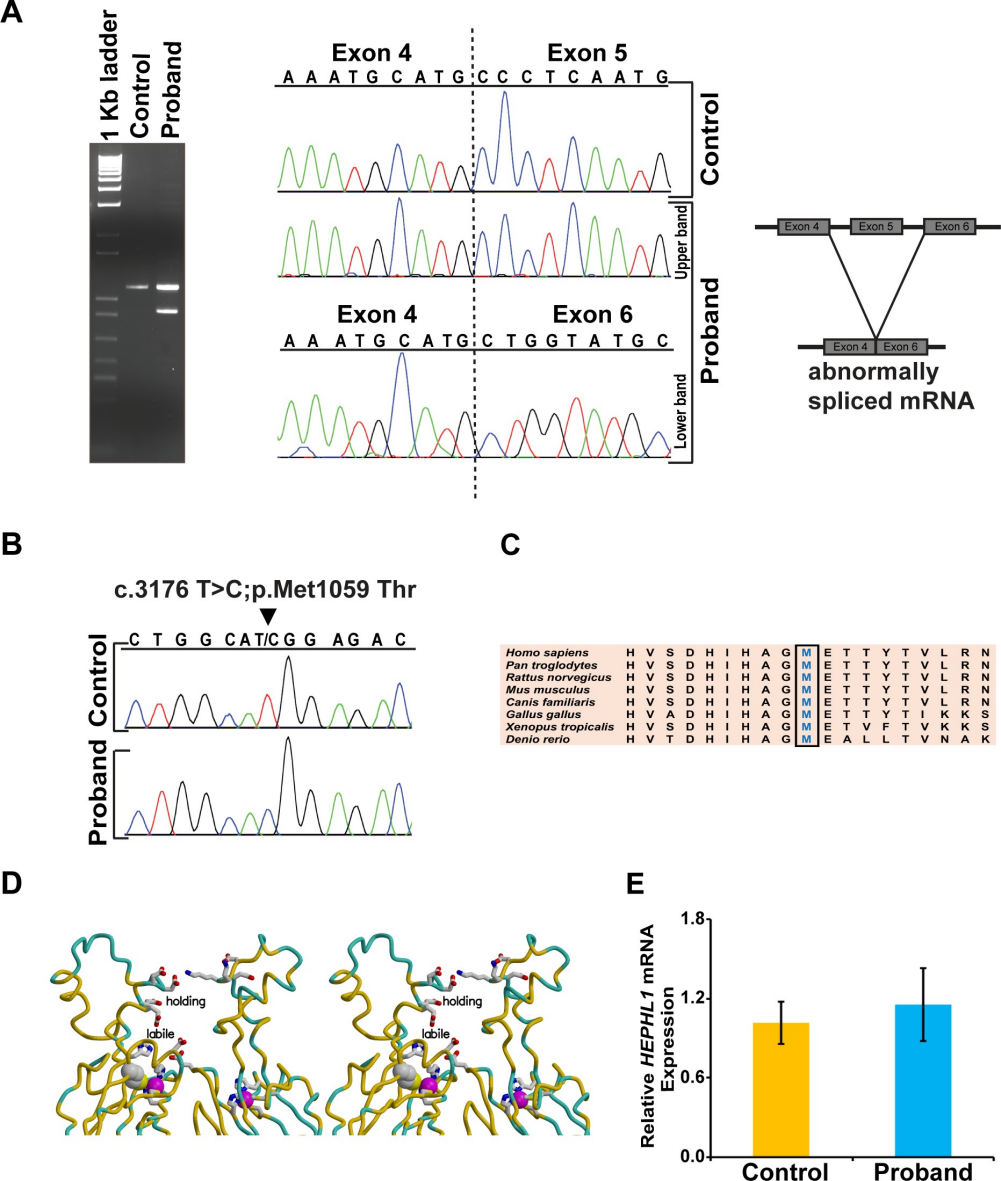
Molecular analyses of *HEPHL1* mutations. (A) Analysis of cDNA from the control and proband. An agarose gel image of the PCR products shows both a correctly spliced and a truncated transcript in the proband. Lane 1 is a 1Kb molecular weight DNA ladder. DNA sequencing of the PCR products confirmed both a correctly spliced (exon 4–5) and a truncated (exon 4–6) transcript in the proband. Skipping of exon 5 in the proband leads to an in-frame deletion of 255 nucleotides. (B) Chromatograms showing presence of c.3176T>C variant in cDNA from the proband. (C) Multiple sequence alignment of HEPHL1 confirms conservation of Met 1059 across different species. (D) Stereo view of portions of domains 2 and 6 of the HEPHL1 model. Met 1059 is shown as space-filling, as are the bound copper ions (purple). Each of these ion binding sites involves the side chains of two histidines, one methionine, and one cysteine. The HEPHL1 main chain is colored by sequence identity to the ceruloplasmin template structure, yellow where identical (52% overall) and light blue where different. Additional side chains that bind the copper ions or surround the “labile” and “holding” sites are shown as bonds, with atoms colored conventionally: carbon, gray; oxygen, red; nitrogen, blue; and sulfur, yellow. (E) Quantitative real time PCR analysis shows no significant difference in mRNA expression between control and patient derived iPSc (values represent mean±SD, n = 6). *GAPDH* was used as a housekeeping gene to normalize the expression of *HEPHL1*.

The maternal variant of *HEPHL1*, a conserved missense mutation in the last nucleotide of exon 5 (c.1063 G>A), is predicted to cause a disruption in splice site function. We found that *HEPHL1* mRNA is expressed in wild type (WT) iPS cells at levels several times greater than those of skin fibroblasts (S1 Fig). Therefore, to explore the effect of the maternal variant on splicing, we performed RT-PCR analysis on control and patient-derived iPSc mRNA samples, using a forward primer that binds to the 5’ UTR and a reverse primer that binds to exon 6. The correctly spliced transcript was present in both control and patient cells (Fig 2A gel, upper band, lane 2 and 3), but the patient’s cells also contained a transcript that spliced directly to exon 6, bypassing exon 5 (Fig 2A, lower band, lane 3). This led to an in-frame deletion of 255 nucleotides (85 amino acids) from the maternal transcript, hence from hereon refereed to as c.809_1063del; p.Leu271_Ala355del. Sequencing of PCR products confirmed these findings (Fig 2A). The 85 amino acids encoded by exon 5 include three critical amino acid residues (H304, C347 and H352) that coordinate with a distal methionine (M357) to constitute a typical type I copper binding site in domain 2; so any resulting translation product would be expected to have altered ferroxidase activity.

To examine the expression of the paternal variant (c.3176T>C), direct sequencing of the PCR product obtained from the RT-PCR reaction was performed using a forward primer in exon 17 and reverse primer in exon 20 (Fig 2B). The mutation affects methionine at a highly conserved position (Fig 2C). Homology modeling using ceruloplasmin as a template (4enz.pdb) shows that an “integral" copper ion is bound to Met 1059, His 1003, His 1054, and Cys 1049 of domain 6; so the mutation to threonine could impair the assembly of this copper-binding site. Ion binding in the nearby sites, referred to as the labile and holding sites [21, 22], may also be influenced by the mutation (Fig 2D).

Next, we compared total *HEPHL1* mRNA levels in control and patient iPSc using quantitative real time PCR analysis and a TaqMan reagent that detected the boundary between exons 11 and 12. We found no significant difference between levels of mRNA expression in control and patient iPSc (Fig 2E), suggesting that the exon skipping in the maternal transcript did not lead to nonsense-mediated decay and that overall expression of the full length paternal and the truncated maternal transcript in the patient was similar to that of wild-type cells. We were unable to analyze HEPHL1 protein status in iPSc or fibroblasts due to the lack of a suitable antibody that can recognize endogenous HEPHL1 protein.

### Each mutation disrupts the ferroxidase activity of HEPHL1

**Fig 3:**
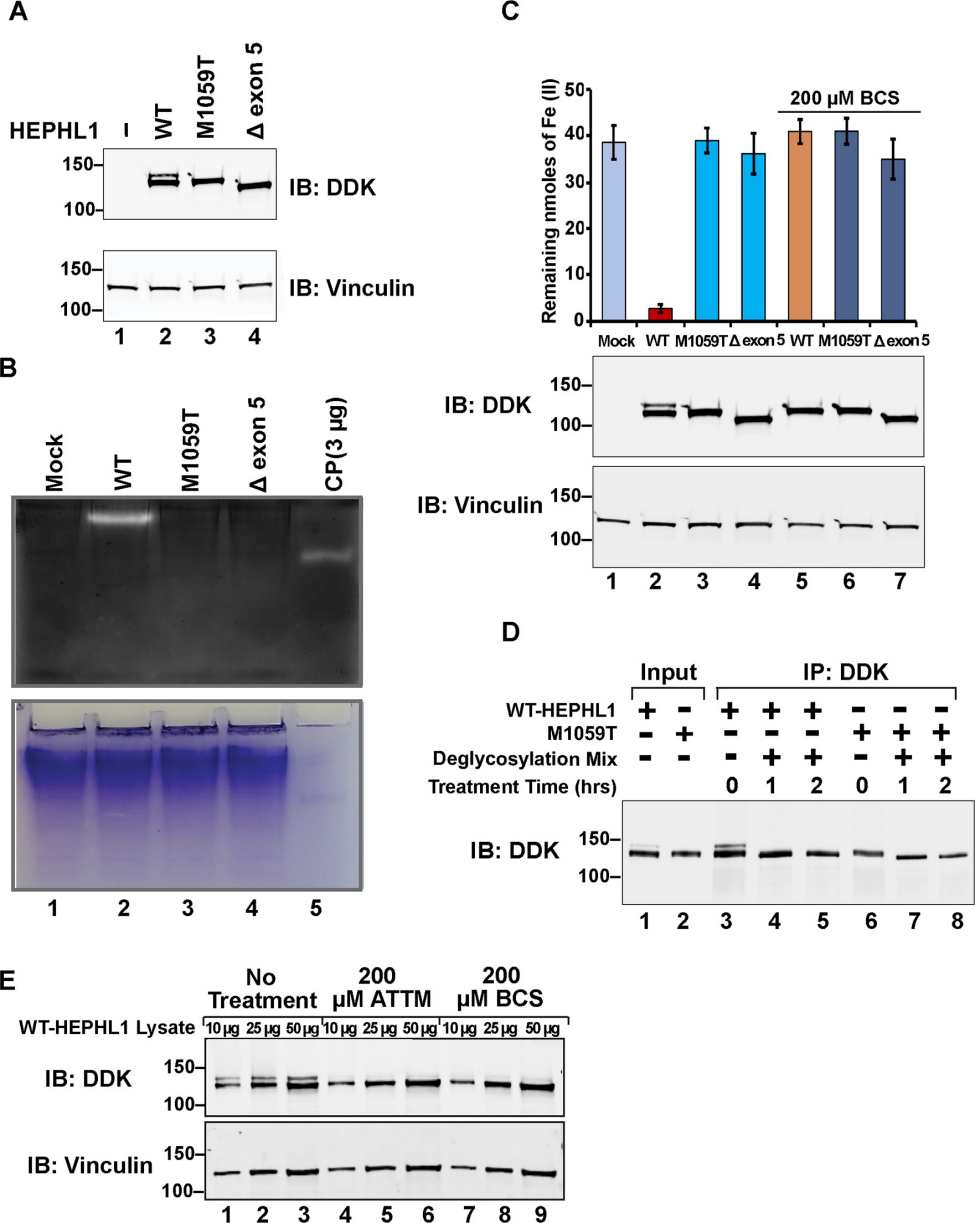
HEPHL1 mutations disrupt copper-dependent ferroxidase activity and glycosylation. (A) Upper panel, immunoblot analysis of whole cell extracts of HEK293 cells overexpressing WT-HEPHL1, HEPHL1 M1059T, HEPHL1 Δexon 5 with anti-DDK antibody. Lower panel shows immunoblotting of whole cell extracts with anti-vinculin antibody to confirm equivalent sample loading. (B) In-gel ferroxidase activity. Whole cell extracts of HEK293 cells overexpressing WT-HEPHL1, HEPHL1 M1059T, HEPHL1 Δexon 5 were prepared under non-denaturing conditions and separated by native gel electrophoresis. After incubation of the gel in ferrous ammonium sulfate solution for 2 h, color was developed with ferrozine. Purified human CP (3 μg) was used as a positive control. The lower panel shows Coomassie blue staining of the gel to confirm equal loading of cellular extracts in lanes 1–4. (C) *In vitro* ferroxidase activity. Anti-DDK beads were used to immunoprecipitate HEPHL1 from whole cell extracts of untreated or cells treated with 200 μM BCS for 2 days. Beads containing immunoprecipitated proteins were incubated with ferrous ammonium sulfate solution followed by incubation in ferrozine solution. Fe (II) forms a complex with ferrozine that can be detected by absorbance at 550 nm. The strong ferroxidase activity of WT-HEPHL1 converted most the Fe (II) to Fe (III), leading to significant reduction in absorbance, while M1059T and Δexon 5 did not show any activity (values represent mean±SD, n = 6). Treatment of cells with 200 μM BCS abrogated ferroxidase activity of WT-HEPHL1, and again no activity was observed for M1059T and Δexon 5 (values represent mean±SD, n = 3). Whole cell extracts used for IP were also immunoblotted with anti-DDK antibody to confirm the expression of HEPHL1 (upper blot). The lower blot shows immunoblotting with anti-vinculin antibody to confirm equivalent sample loading. (D) HEPHL1 is glycosylated. Whole cell extracts of HEK293 cells overexpressing WT-HEPHL1 or HEPHL1 M1059T were immunoprecipitated using anti-DDK beads. Beads were treated with deglycosylation mix for the indicated time points and processed for immunoblotting with anti-DDK antibody. The deglycosylation treatment removed the upper band. (E) Copper is required for HEPHL1 post-translational modification. HEK293 cells were transfected with WT-HEPHL1 and treated with the copper chelators ATTM or BCS. Immunoblotting of lysates with anti-DDK antibody shows that the high molecular weight form of HEPHL1 was diminished when copper was chelated.

HEPHL1 is predicted to be a copper-dependent ferroxidase due to its similarity in sequence and putative structure to the known ferroxidases, CP and HEPH [15]. Since both patient *HEPHL1* mutations are predicted to disrupt type I copper binding sites, we hypothesized that ferroxidase activity would be severely reduced. Our attempts to measure endogenous ferroxidase activity of HEPHL1 in iPSc or fibroblasts cell lines failed, likely because baseline ferroxidase activity is below the limit of detection in these cells. To circumvent this problem, we performed overexpression studies in HEK293 cells using a WT-HEPHL1 construct and constructs for the paternal (HEPHL1 M1059T) and maternal (HEPHL1 Δexon 5) alleles. These were created by site-directed mutagenesis using a commercially available *HEPHL1* expression vector with a C-terminal myc-DDK tag (Origene, Rockville, MD) as template. Transfection of these constructs into HEK293 cells resulted in robust expression, as illustrated by western blot analysis using anti-DDK antibody (Fig 3A, lanes 2–4). To measure HEPHL1 ferroxidase activity, we prepared protein extracts under native conditions from the overexpressing HEK293 cells. Equivalent amounts of protein extracts were then separated by nondenaturing gel electrophoresis followed by an in-gel ferroxidase assay. After incubation of the gel in saturated ferrous ammonium sulfate solution for 2 h, the gel was incubated with a ferrozine solution. Ferrozine turns red-purple when Fe (II) is bound. Oxidation of Fe (II) to Fe (III) indicates ferroxidase activity and can be seen by the formation of a discrete clear area (band) in the gel. As shown in Fig 3B, a discrete band was observed in WT-HEPHL1 lane, suggestive of active ferroxidase; the level was slightly higher than that attributed to 3 μg of purified ceruloplasmin, a known ferroxidase (lane 5). In contrast, we did not observe any ferroxidase activity when HEPHL1 M1059T or HEPHL1 Δexon 5 were expressed (Fig 3B, lanes 3 and 4).

It remained possible, however, that HEPHL1 M1059T or HEPHL1 Δexon 5 retained some low level of ferroxidase activity. Therefore, we developed a simple and quantitative ferrozine-based colorimetric assay to quantitatively assess ferroxidase activity by the conversion of Fe (II) to Fe (III) (S2 Fig). We used this assay to measure ferroxidase activity in lysates of HEK293 cells expressing HEPHL1 constructs. Expression of WT-HEPHL1 led to a significant reduction in the absorbance of the Fe (II)–ferrozine complex as compared to the mock-transfected cell lysate (Fig 3C), reflecting significant ferroxidase activity. Consistent with the results of the in-gel assay, expression of either HEPHL1 M1059T or HEPHL1 Δexon 5 resulted in non-detectable conversion of Fe (II) to Fe (III). In fact, the Fe (II)–ferrozine absorbance was identical to that of mock-transfected cells, indicating that both mutants were catalytically inactive in the assay. To confirm that the inability of HEPHL1 M1059T and HEPHL1 Δexon 5 to oxidize Fe (II) was not simply due to lack of expression, we carried-out an anti-DDK western blot on lysates. As shown in lower panels of Fig 3C, WT-HEPHL1, HEPHL1 M1059T and HEPHL1 Δexon 5 were robustly expressed at comparable levels. Taken together, these results provide strong evidence that the paternal and maternal mutations in HEPHL1 each completely abolish ferroxidase activity.

### Glycosylation and ferroxidase activity of HEPHL1 is copper dependent

Immunoblot analysis of lysates from HEK293 cells transfected with WT-HEPHL1 consistently identified an additional, higher molecular weight species, presumably a post-translationally modified form of HEPHL1 (Fig 3A, lane 2 and Fig 3C, lane 2). This form was lost in HEPHL1 M1059T and HEPHL1 Δexon 5 (Fig 3A, lanes 3 and 4, Fig 3C, lanes 3 and 4), suggesting that these mutations interfere with the post-translational modification of HEPHL1 that, as shown previously for HEPH, likely involves glycosylation [23]. To explore this, we immunoprecipitated WT-HEPHL1 and HEPHL1 M1059T using anti-DDK beads and treated with glycosidases that cleaved both *N*-linked and *O*-linked glycans. As shown in Fig 3D, the higher molecular weight form of WT-HEPHL1 completely disappeared after the treatment, indicating that this higher molecular weight form is glycosylated HEPHL1 (Fig 3D, lanes 3–5). Treatment with glycosidases also slightly increased the electrophoretic migration of HEPHL1 M1059T (Fig 3D, lanes 6–8) suggesting that HEPHL1 M1059T may also be glycosylated, albeit to a lesser extent. As further evidence for HEPHL1 glycosylation, mass-spectrometry analysis revealed that WT-HEPHL1 is *N*-linked glycosylated at three conserved asparagines (N^161^YT, N^407^AS, N^772^RT), while HEPHL1 M1059T is glycosylated at only two of these sites (N^161^YT, N^772^RT). Interestingly, the Δexon 5 mutant failed to show glycosylation at any of the three sites (S3 Fig). Additionally, we detected two *O*-linked glycosylation sites in WT-HEPHL1 (S1076 and T1077), but glycosylation of neither site was affected in HEPHL1 M1059T and HEPHL1 Δexon 5.

To determine whether copper might be required for HEPHL1 *N*-linked glycosylation, we transfected HEK293 cells with the WT-HEPHL1 construct and treated the cells over a 2-day period with either ammonium tetrathiomolybdate (ATTM) or bathocuproinedisulfonic acid (BCS) to deplete copper. Using three different amounts of untreated, ATTM-treated, and BCS-treated cell lysates, we ran an anti-DDK western blot. As shown in Fig 3E, untreated WT-HEPHL1 appeared in both the unmodified and the high molecular weight glycosylated forms (lanes 1–3), but treatment of cells with ATTM (lanes 4–6) or BCS (lanes 7–9) markedly reduced the higher molecular weight, glycosylated form. The lower, un-modified form of HEPHL1 remained unchanged. These results suggest that copper is required for maintaining HEPHL1 in the mature glycosylated form, whether by fostering glycosylation or by inhibiting deglycosylation.

Several studies have shown the importance of copper for the enzymatic activity and/or structural integrity of HEPH and CP [23, 24]. Since both paternal and maternal mutations affect the residues involved in type I copper binding sites in HEPHL1, we investigated whether decreased copper availability leads to impaired activity of HEPHL1. We therefore transfected HEK293 cells with WT-HEPHL1, HEPHL1 M1059T or HEPHL1 Δexon 5 constructs and treated the cells with BCS. WT-HEPHL1 transfected cells that were treated with BCS had the same ferroxidase activity as for mock transfected HEK293 cells (Fig 3C, BCS treatment), and significantly lower than its activity in the absence of BCS, suggesting that copper depletion eliminated the ferroxidase activity. As expected, no further change in ferroxidase activity was found in either HEPHL1 M1059T or HEPHL1 Δexon 5 transfected cells upon treatment of cells with BCS. Taken together, these results indicate that the ferroxidase activity seen in HEPHL1 is copper-dependent.

### Increased iron and reduced lysyl oxidase activity in patient fibroblasts

**Fig 4:**
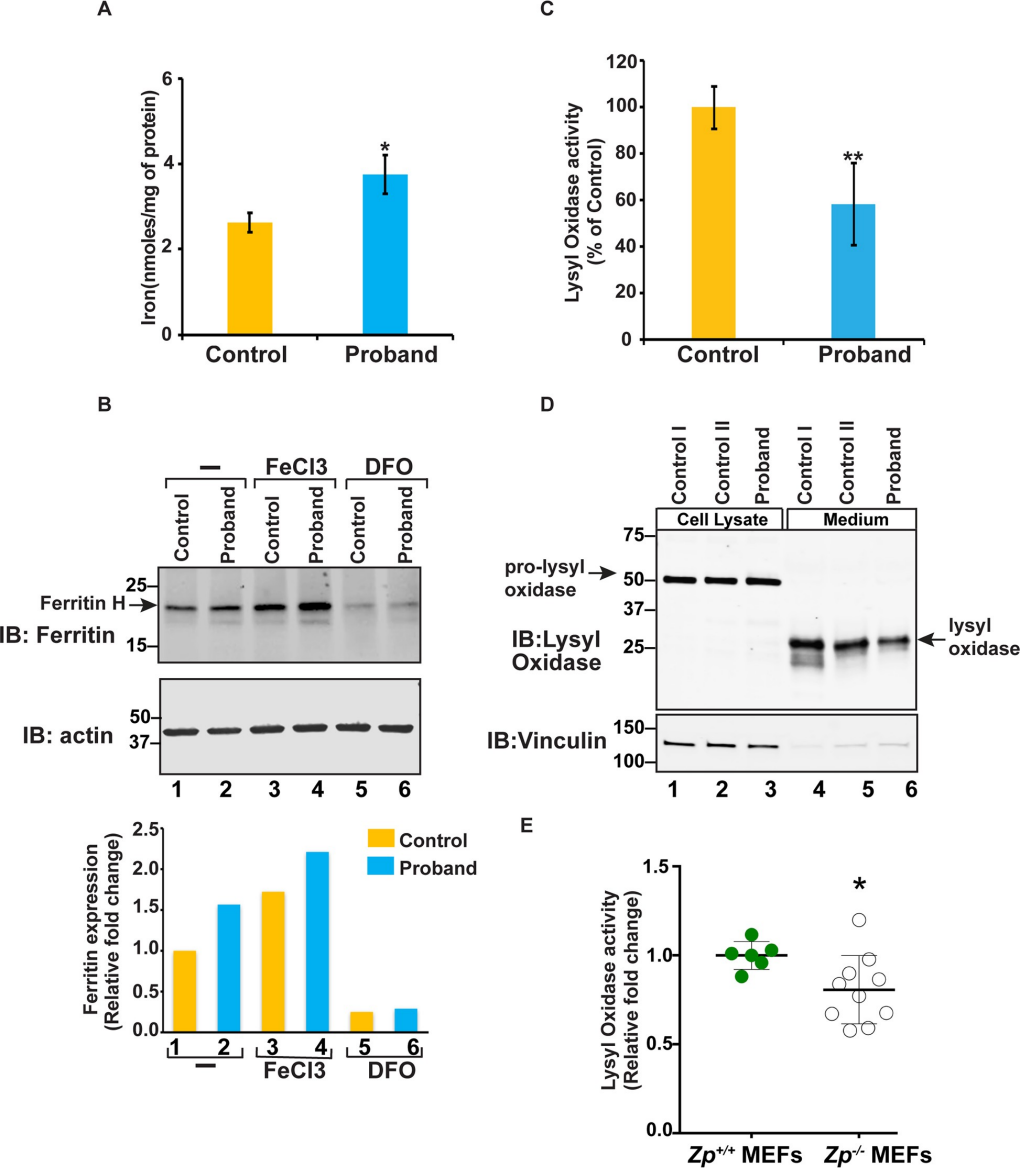
Functional consequences of the loss of HEPHL1. (A) Increased total intracellular iron content in the patient’s fibroblasts compared to control. The control value represents the mean of data from two independent normal cell lines (error bars indicate ± SD, n = 8, * *p*<0.05). (B) Upper panels showing a representative anti-ferritin immunoblot of whole cell extracts of fibroblasts either untreated or treated with either 100 μM FeCl_3_ or 100 μM DFO (top). Anti-ß actin immunoblot shows equivalent loading of lysates (bottom). Lower panel shows fold changes in expression, calculated by quantitative analysis of bands using image studio software (Li-COR Biosciences). (C) Reduced lysyl oxidase enzyme activity in the patient’s fibroblast. Lysyl oxidase activity was measured using a fluorescent enzyme assay. Each sample was measured in triplicate, and enzyme activity was normalized to total cellular protein content for each sample. Results are expressed as percentage of lysyl oxidase activity in patient’s fibroblasts relative to control. Control represents mean value of four independent normal cell lines (error bars indicate ± SD, n = 13, **p<0.0001). (D) Reduced levels of secreted lysyl oxidase in patient’s fibroblasts. Equal amounts of cell culture medium from two controls and patient’s fibroblasts grown to confluency were collected and concentrated using a protein concentrator column. Whole cell lysates were prepared by lysis of cells in Triton X-100 buffer. The upper panel shows a representative anti-lysyl oxidase immunoblot of whole cell extracts (lanes 1–3) and cell culture medium (lanes 4–6). The lower panel shows anti-Vinculin immunoblot to confirm equal loading of lysates in lanes 1–3. (E) Reduced lysyl oxidase activity in *Zp*^*-/-*^ MEFs. Three *Zp*^*+/+*^ and five *Zp*^*-/-*^ MEF cell lines were used to measure lysyl oxidase activity in two independent experiments. Each sample was measured in triplicate, and enzyme activity was normalized to total cellular protein content for each sample. Results are expressed as relative fold changes in lysyl oxidase activity. Data are shown as mean ± SD, n = 6 for *Zp*^*+/+*^ and n = 10 for *Zp*^*-/-*^,* *p*<0.05.

Given that the patient mutations abrogated the ferroxidase activity of HEPHL1, we assessed the basal intracellular iron content in dermal fibroblasts. The mutant fibroblasts showed a statistically significant increase in basal iron content compared to that of control fibroblasts (Fig 4A). Western blot analysis also showed an increased expression of ferritin in patient cells (Fig 4B, lanes 1 and 2). Treatment of fibroblasts with FeCl_3_ led to a further increase in ferritin expression with more pronounced accumulation in patient cells (Fig 4B, lanes 3 and 4). Treatment of cells with the iron chelator deferoxamine (DFO) reduced the ferritin expression in both control and patient fibroblasts (Fig 4B, lanes 5 and 6), consistent with the modulation of ferritin expression by intracellular iron content.

We were curious if impaired ferroxidase activity of HEPHL1 also influences the activity of copper dependent enzymes. We therefore measured the activity of the lysyl oxidase (LOX) enzyme, which requires copper [Cu (II)] for enzymatic activity. To our surprise, lysyl oxidase activity in the patient's fibroblasts was significantly reduced compared to unaffected controls (Fig 4C). To further explore this phenomenon, we analyzed the two different forms of the LOX protein (i.e., the immature prolysyl oxidase and the mature, active enzyme) in whole cell extracts and the medium collected from confluent cultures of the patient’s and control fibroblasts. Immunoblot analysis using an antibody that detects all the forms of LOX showed no difference in the levels of the 52 kDa prolysyl oxidase in whole cell extracts (lanes 1–3). However, levels of the 29 kDa, catalytically functional LOX were significantly decreased (by 37%) in the patient’s fibroblast medium compared to that of two unaffected controls (lanes 4–6). This finding suggests that reduction in the levels of lysyl oxidase and the corresponding enzymatic activity in the patient’s fibroblasts were due to altered post-translational processing of this enzyme in the absence of functional HEPHL1.

### Genetic ablation of *Hephl1* in mice leads to a curly whisker phenotype

**Fig 5:**
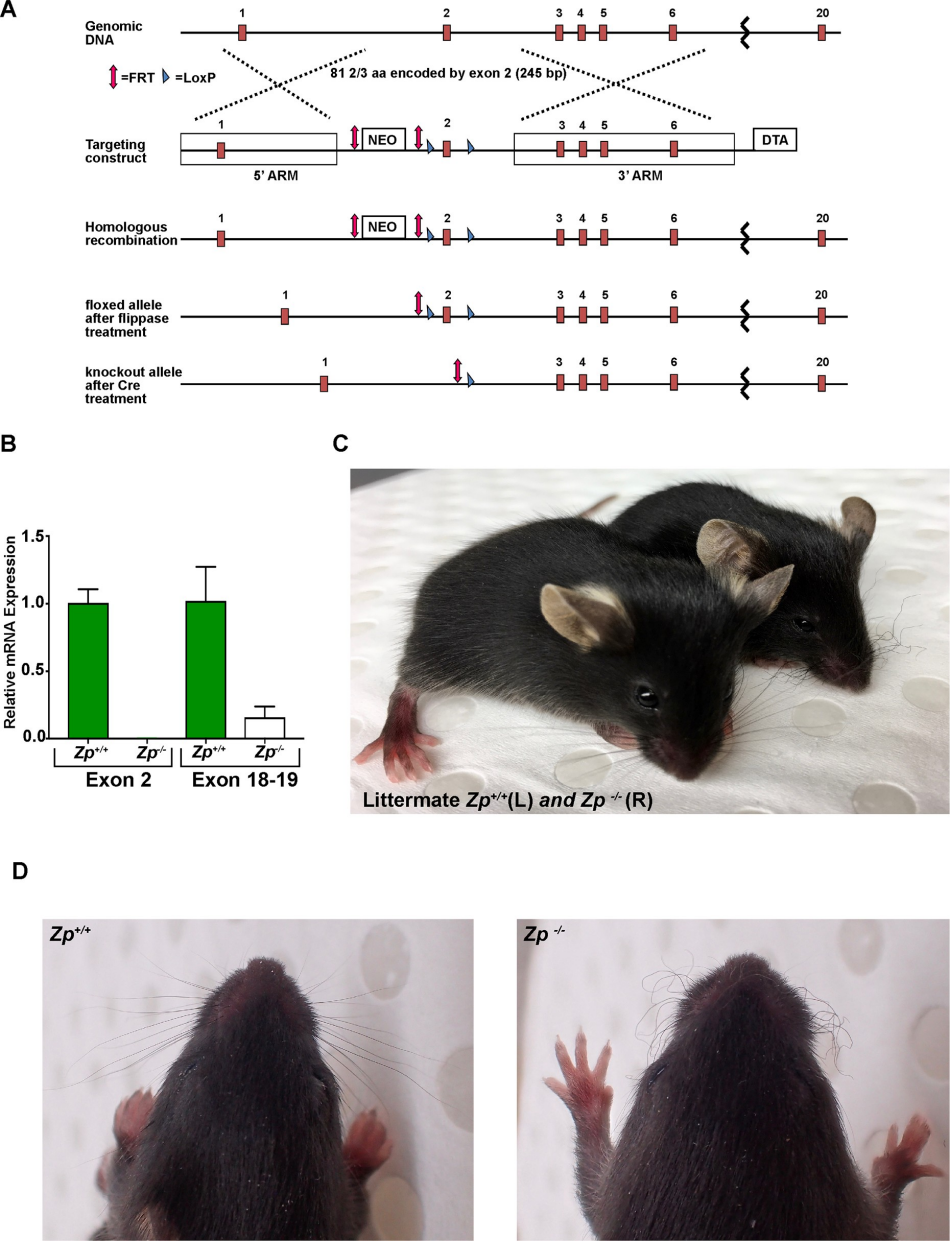
Generation and phenotype of *Hephl1* knockout mice. (A) Schematic showing structure of the *Hephl1* genomic region and the strategy used to generate the *Hephl1* knockout (*Zp*^*-/-*^) allele (for details see Materials and Methods). (B) Quantitative real time PCR analysis of *Hephl1* mRNA expression in *Zp*^*+/+*^ and *Zp*^*-/-*^ placental tissue. No expression was observed in *Zp*^*-/-*^ placental tissue using primers specific for exon 2. Expression was significantly reduced in *Zp*^*-/-*^ when primers specific for a region downstream of knockout site (exon 18–19) were used, indicating instability of transcript lacking exon 2. *Hprt* was used as a housekeeping gene to normalize the expression of *Hephl1*. (C) Phenotype of the *Zp*^*-/-*^ mouse showing short, curled whiskers (vibrissae). A wild-type (*Zp*^+/+^) littermate is shown on the left. (D) A close-up photograph of whiskers.

To explore the physiological consequences of loss of HEPHL1 activity, we generated *Hephl1* knockout (*Zp*^*-/-*^) mice. Exon 2 of the *Hephl1* gene was chosen for gene targeting because it is located near the start of the protein-coding region and encodes residues that make up the Type II copper binding site required for ferroxidase activity. Removal of exon 2 (245 bp) also leads to a frameshift that introduces an early stop codon. Quantitative PCR analysis of *Hephl1* mRNA extracted from *Zp*^*+/+*^ and *Zp*^*-/-*^ placental tissues confirmed the loss of exon 2. In addition, only residual expression of downstream exons was detected (Fig 5B), indicating instability of the *Hephl1* transcript lacking exon 2. Phenotypically, *Zp*^*-/-*^ mice were viable and bred successfully, and the genotype ratios in the progeny were consistent with normal perinatal viability. All mice with ablation of *Hephl1* exhibited short, curled whiskers (vibrissae) throughout life (Fig 5C and 5D). Heterozygous mice (*Zp*^*+/-*^) did not exhibit the curly whisker phenotype, indicating that this phenotype is recessive. Although a full characterization of these mice was beyond the scope of the present study, we investigated whether the lysyl oxidase abnormalities we observed in the patient’s fibroblasts are recapitulated in our mouse model. We established mouse embryonic fibroblasts (MEFs) cultures from *Zp*^*+/+*^ and *Zp*^*-/-*^ embryos and measured lysyl oxidase activity. Consistent with the results in patient’s fibroblasts, we also found a significant reduction in the activity of lysyl oxidase in *Zp*^*-/-*^ MEFs (Fig 4E). Taken together, our results strongly suggest that lysyl oxidase activity is regulated by HEPHL1.

## Discussion

The identification of biallelic mutations (p. Leu271_ala355del and p. Met1059Thr) in the *HEPHL1* gene of a 5-year-old boy prompted us to investigate the function of normal and mutant HEPHL1 proteins. First, we examined the copper-binding sites. A unique characteristic of multi-copper oxidases is the presence of at least one of each of three types of copper binding sites: type I, type II and binuclear type III. A type I copper site accepts electrons from the substrate while the trinuclear cluster, comprising a type II and a binuclear type III site, operates as a center where dioxygen is reduced to two water molecules [9]. CP, a six-domain multi-copper oxidase, contains three type I copper sites (in domains 2, 4 and 6) and a trinuclear cluster at the interface of domains 1 and 6 [25]. Based on the known crystal structure of CP, homology modeling indicates that all type I, II, and III copper sites for the 6 copper ions in CP are also present in HEPHL1 [15]. In the patient, both the paternal and maternal mutations affect residues involved in the binding of copper at type I sites, which leads to loss of ferroxidase activity. A highly conserved methionine involved in the patient’s paternal mutation (M1059) is part of the most essential type I copper binding site that is couple to the trinuclear cluster in domain 6. The maternal deletion of exon 5 removes 85 residues in domain 2 of HEPHL1, including three residues (H304, C347, H352) that coordinate with a distal methionine (M357) to constitute a typical type I site. For CP, while the type I site in domain 2 is atypical [25], a mutation at this site was shown to prevent copper incorporation, suggesting that this domain 2 site is critical for the ferroxidase function of CP [24]. The deletion of much of domain 2 in HEPHL1 resulting from the maternal mutation presumably disrupts the overall protein structure, eliminating copper binding in domain 2 and quite possibly elsewhere.

We consistently found that wild-type-HEPHL1 appeared as two separate bands, with the higher molecular weight band presumably representing a post-translationally modified form of HEPHL1. For the structurally similar ferroxidase HEPH, the higher molecular weight form reflects glycosylation of the protein. Transfection experiments performed in polarized differentiated T84 cells and subsequent pulse-chase analysis revealed that, although HEPH is synthesized as a single chain polypeptide of 144 kDa, it runs as a double band because the newly synthesized HEPH is modified to a mature 161 kDa species by *N*-linked glycosylation [23]. Similarly, in this paper, we show that the higher molecular weight form of HEPHL1 (Fig 3D) is glycosylated. Whereas previous studies have revealed that the percentage of glycosylated HEPH was higher than the non-glycosylated form [23], we observed a smaller percentage of the glycosylated form of WT-HEPHL1 compared to the non-glycosylated form. This may indicate that transiently expressed proteins have not reached full maturation after 48 h. Interestingly, we found that the glycosylation and ferroxidase activity of HEPHL1 is copper-dependent. Using HEK293 cells overexpressing WT-HEPHL1, we showed that copper is necessary for the subsequent glycosylation and formation of mature catalytically active holoenzyme. The chelation of copper with BCS abrogated both glycosylation and ferroxidase activity, likely due to rapid degradation of the unstable apo-HEPHL1 moiety. Further studies will be needed to determine the precise function of glycosylated and non-glycosylated forms of HEPHL1.

These *HEPHL1* mutations have functional consequences. First, both the paternal (M1059T) and maternal (Δexon 5) mutants, which involve copper binding sites, showed complete lack of ferroxidase activity. This was associated with a modest but significant increase in intracellular iron content and ferritin expression in the patient’s cultured fibroblasts (Fig 4A and 4B), supporting a role for HEPHL1-mediated ferroxidase activity in modulating intracellular iron content. Although HEPHL1 does not appear to be the principal ferroxidase in blood, and the patient’s serum iron was 107 μg/dL (normal, 30–110), HEPHL1-mediated ferroxidase activity could play a more specific role in certain tissues or cells, where loss of its activity cannot be fully compensated by other ferroxidases. Analysis of mRNA expression suggests that HEPHL1 is robustly expressed in rodent placenta and embryo and at a lower level in heart and kidney, while no expression was found in liver or enterocytes. At the protein level, HEPHL1 was detected in rat placenta, mouse mammary tissue and whole embryo, as well as BeWo, MCF7 and T47D cell lines. Interestingly, no protein expression of HEPHL1 was detected in mouse serum or enterocytes, where CP and HEPH, respectively, are prominently expressed [15]. This distribution suggests a tissue-specific requirement for ferroxidases.

Another function of HEPHL1 is likely manifest in the hair follicle, which depends upon transition metals for its structure [26]. A recent genome-wide transcriptome analysis by deep RNA-sequencing identified *Hephl1* as a signature gene of the mouse hair follicle’s transient amplifying cells (HF-TACs) from postnatal day P(5) back skin [27]. Interestingly, expression of *Hephl1* in TACs is significantly higher than both *Heph* and *Cp* (www.hair-gel.net), suggesting a unique role for *Hephl1* in these specialized progenitor cells in regulating hair follicle morphogenesis. Indeed, our patient with biallelic *HEPHL1* mutations displayed sparse, twisted, brittle and easily broken scalp hairs, with similar findings in the eyebrows and eyelashes. The boy’s hair also exhibited two different structural abnormalities, i.e., trichorrhexis nodosa (broken, nodular shafts) and pili torti. Pili torti (hair twisted) is a rare, congenital or acquired condition characterized by twisted and flattened hair shafts at irregular intervals, caused by changes in cell shape and thickness of both the outer and inner root sheaths [28]. The Online Mendelian Inheritance in Man (OMIM) database identified 24 entries for pili torti illustrating its association with a wide spectrum of neurological disorders including Björnstad syndrome [29] (MIM 262000), ectodermal dysplasias such as Rapp-Hodgkin syndrome [29] (MIM 129400) as well as Menkes disease [29] (MIM 309400), also known as kinky hair syndrome [30, 31]. We also note that Belted Galloway cattle with homozygous loss-of-function mutations in *HEPHL1* have hypotrichosis [32], and our *Hephl1* knockout mouse exhibits curly whiskers resembling pili torti.

A third function of HEPHL1 could be to serve as a cuprous oxidase, catalyzing the conversion of Cu (I) to Cu (II). Other ferroxidases such as bacterial CueO, yeast Fet3p and human CP also exhibit cuprous oxidase activity, using Cu (I) as substrate [33–36]. Kinetic analysis of Fet3p showed that both ferroxidase and cuprous oxidase reactions are catalyzed at the same type I copper site [35]. Along with the data presented here showing that the patient’s mutations completely abrogated ferroxidase activity, this raises the possibility that HEPHL1 mutations might also interfere with the cuprous oxidase activity of the enzyme. The end result would be reduced availability of Cu (II) for lysyl oxidase. Indeed, we found that the levels of the catalytically active form of lysyl oxidase (Cu (II) bound holoenzyme) and its corresponding enzymatic activity were significantly diminished in fibroblasts from our patient. Consistent with this analysis, we also observed a significant reduction in lysyl oxidase activity in MEFs derived from *Hephl1* knockout embryos. Taken together, these results provide an insight into a possible physiological role of HEPHL1 in regulating the activity of lysyl oxidase and likely other cuproenzymes.

In summary, we have established HEPHL1 as a physiologically important ferroxidase whose deficiency in a human, as in animals, results in structural hair abnormalities. The patient's mutations lead to abolished ferroxidase activity, and the patient's cultured fibroblasts exhibited increased iron accumulation and reduced amounts of mature, secreted lysyl oxidase and corresponding enzymatic activity. Of interest, we observed increased joint mobility in our patient, which could be a consequence of decreased lysyl oxidase activity causing lax connective tissue. We do not know if our patient’s mild cognitive delays, heat intolerance, and chronic leg pain are related to the HEPHL1 mutations. Identification of additional patients with HEPHL1 loss-of-function mutations should shed further light on the clinical phenotype associated with this condition, and the *Hephl1* knockout mouse can be used to further investigate HEPHL1 function.

## Materials and methods

### Ethics statement

The patient and family members were enrolled in the National Institutes of Health Undiagnosed Diseases Program (UDP) [37–39] and in protocol 76-HG-0238, “Diagnosis and Treatment of Patients with Inborn Errors of Metabolism and Other Genetic Disorders” approved by the Institutional Review Board (IRB) of the National Human Genome Research Institute (NHGRI). Written informed consent was obtained, and the patient was evaluated at the NIH Clinical Center.

All work performed on mice was in accordance with the National Institutes of Health (NIH) guidelines, as described in the Guide for the Care and Use of Laboratory Animals of the NIH, and with approval from the Office of Laboratory Animal Care at the University of California, Berkeley, and the QIMR Berghofer Medical Research Institute Animal Ethics Committee.

### Whole exome and Sanger sequencing

Genomic DNA was extracted from blood leukocytes following standard protocols. Whole exome sequencing (WES) was performed using an Agilent 38MB Sure Capture System. Image analysis and base calling were performed using Illumina Genome Analyzer Pipeline software (versions 1.13.48.0) with default parameters. Reads were aligned to a human reference sequence (UCSC assembly hg19, NCBI build 37) using the Efficient Large-scale Alignment of Nucleotide Databases (Illumina, San Diego, CA, USA) package. Genotypes were called at all positions where there were high-quality sequence bases using the Most Probable Genotype Bayesian algorithm [40], and variants were filtered using the graphical software tool VarSifter v1.5 [41]. Validation of *HEPHL1* sequence variants and segregation with disease were confirmed by Sanger sequencing. Genomic DNA flanking the site of paternal or maternal variants was amplified with HotStar Taq DNA polymerase (Qiagen, Valencia, CA) using the following primer sets: set 1 paternal, CTTTCCTGGGACATTCCAAA and TCCTGTTTTGGGGGTCTACA; set 2 maternal, CCAGCCACCTTCCTTACAAC and TGAGCACTAGTGACTGTGTGGTT. PCR conditions were: initial denaturation at 95°C for 5 min, followed by 35 cycles of denaturation at 95°C for 30 s, annealing at 55°C for 30 s, extension at 72°C for 30 s, followed by a final extension step at 72°C for 5 min. PCR products were purified using ExoSAP-IT (Affymetrix) and sent to the Macrogen service center (Macrogen USA, Rockville, MD) for sequencing. Sequencing files were evaluated using Sequencher v5.0 software (Gene Codes Corporation, Ann Arbor, MI).

### Fibroblasts and iPSc

Primary dermal fibroblasts from the patient were derived from a forearm skin biopsy and cultured in DMEM supplemented with 10% fetal bovine serum (Thermo Fisher Scientific, Waltman, MA), MEM nonessential amino acid solution (Sigma-Aldrich, St. Louis, MO), penicillin–streptomycin and 2 mM L -glutamine (Sigma-Aldrich). Control fibroblasts were obtained from the ATCC (PCS-201-012, Manassas, VA, USA) and Coriell Institute of Medical Research (GM08398, GM00942 Camden, New Jersey). Induced pluripotent stem (iPS) cell derivation and characterization using early passage dermal fibroblasts from the patient were done at ALSTEM (ALSTEM cell Advancements, Richmond, CA) by ectopic expression of OCT4, SOX2, KLF4, and c-MYC genes using episomal plasmids. Routine culture of iPS cells was performed using mTesR1 with 5X Supplement (STEMCELL Technologies, CA) as described in detail under ALSTEM protocol iPS11 (www.alstembio.com/protocols). All cell lines were grown in a 37^o^ C incubator with 5.0% CO_2._

### RNA extraction, PCR and quantitative PCR

Total RNA was extracted from control and patient cells using a RNeasy Mini Kit (Qiagen). Genomic DNA contamination was removed using the Turbo DNA free kit (Thermo Fisher Scientific) following the manufacturer’s instructions. Single-stranded cDNA was synthesized using the High Capacity RNA-to-cDNA kit (Applied Biosystems). To analyze *HEPHL1* mutations at the cDNA level, PCR amplifications of the control and patient cDNA were carried out using forward primer CTGCTTGAGTTACATCCACAA and reverse primer TGACCCTTCATCTTGGGGTA for the maternal variant c. G1063A, and forward primer CTTTCCTGGGACATTCCAAA and reverse primer GACCCAGATTCTTGCCAAAG for the paternal variant c. T3176C. PCR products were separated on a 1% agarose gel and extracted using a gel extraction kit (Qiagen). Direct sequencing of the PCR products was carried out by Sanger sequencing as described above. For relative quantitation of the *HEPHL1* transcript levels, real time PCR was performed with an ABI7300 Genetic Analyzer (Applied Biosystems) using the FAM-labeled TaqMan Probe and primers mix (Thermo Fisher Scientific, HS01376171_m1); the primers anneal to the junction between exon11/exon 12 in *HEPHL1*. Gene expression values were normalized to the expression of the reference gene glyceraldehyde-3-phosphate dehydrogenase (*GAPDH*) (Thermo Fisher Scientific Hs03929097_g1).

### Homology modeling

A three-dimensional model of human HEPHL1 was built based on its homology to ceruloplasmin, using Prime software tools (Schrodinger, LLC). Of the 1043 HEPHL1 residues modeled, ranging from Thr 26 to Asn 1068, 541 (52%) are identical to the nearest residue in the ceruloplasmin template structure 4enz.pdb [22]. The model was rendered using the programs MolScript [42] and Raster3D [43].

### Expression vectors and site-directed mutagenesis

The pCMV6 plasmid containing the entire sequence of the human HEPHL1 ORF (NM_001098672) with a C-terminal MYC-DDK tag [myc-DDK-HEPHL1, hereafter WT (wild type)-HEPHL1] was obtained from Origene (RC214648, Origene Technologies, Rockville, MD, USA). The Q5 Site-Directed Mutagenesis Kit (New England Biolabs) was used to generate both the maternal expression construct lacking exon 5 (myc-DDK-HEPHL1 Δexon 5, hereafter HEPHL1 Δexon 5) and the paternal expression construct (myc-DDK-HEPHL1 M1059T, hereafter HEPHL1 M1059T) using the manufacturer’s protocol. pCMV6-Entry, an empty vector with a C-terminal Myc-DDK Tag (PS100001, Origene Technologies) was used as a control/mock vector in the transfection assays. All constructs were verified by DNA sequencing.

### Transfection and immunoblotting

HEK293 cells in the exponential growth phase in 10 cm culture dishes were transiently transfected with 10–12 μg of WT-HEPHL1, HEPHL1 Δexon 5, or HEPHL1 M1059T constructs using Lipofectamine 2000 reagent (Thermo Fisher Scientific) according to the manufacturer’s instructions. Cells were incubated for 48 h with a change of medium the day after transfection. For copper-chelation, 200 μM of either ammonium tetrathiomolybdate (ATTM) (Sigma-Aldrich) or 200 μM bathocuproinedisulfonic acid (BCS) (Sigma-Aldrich) was added to the medium immediately after transfection, and the medium (with drug) was changed the day after transfection. Cells were collected, washed twice with PBS and lysed using native lysis buffer (Abcam, Cambridge, MA) with Protease (Roche) and Phosphatase (Roche) Inhibitors. Lysates were kept on ice for 30 min followed by mechanical shredding by passing through a 25 5/8 G needle 5–7 times. After centrifuging to pellet insoluble material, supernatants were transferred to a new tube and protein concentrations were measured using the DC protein assay (Bio-Rad, Hercules, CA). To each sample, equal amounts of 2X Laemmli Sample Buffer (Bio-Rad) with beta-mercaptoethanol were added before boiling at 95°C. Samples were then fractionated on 4–15% Mini-Protean TGX Stain Free Gel (Bio-Rad) and run at 150V, followed by transfer onto a low fluorescence PVDF or nitrocellulose membrane using the Trans-Blot Turbo system (Bio-Rad). Membranes were blocked in Odyssey blocking buffer (LI-COR) for 2 h at room temperature and probed with an anti-DDK mouse monoclonal antibody (Origene TA50011-100, Clone OTI4C5) overnight at 4°C. Membranes were then washed with TBST (Tris-buffered saline with 0.05% Tween 20) followed by incubation with IRDye 800 CW or IRDye 680RD goat anti-mouse secondary antibody (LI-COR Biosciences) for 1 h at room temperature. Images were captured using the LI-COR Odyssey CLx imaging system. Mouse monoclonal anti-Vinculin (V9131, Sigma-Aldrich) was used to confirm equivalent sample loading.

### Measurement of ferroxidase activity

Lysates were prepared from transiently transfected HEK293 cells with WT-HEPHL1, HEPHL1 Δexon 5, or HEPHL1 M1059T constructs as described above for immunoblot analysis. To visualize *in-vitro* ferroxidase activity, equal amounts of 2X native PAGE sample buffer (Bio-Rad) were added to lysates and samples were fractionated on 4–15% Mini-Protean TGX Stain Free Gels (Bio-Rad) at a constant 100 V for 21 min, then at 33 V for 7 h, using native PAGE running buffer (Bio-Rad). Gels were then incubated at 37°C for 2 h in 0.00784% ferrous ammonium sulfate [Fe(NH_4_)_2_(SO_4_)_2_ 6H_2_O] solution prepared in 0.1 M sodium acetate, pH 5.0. After incubation, gels were placed in 15 mM ferrozine Solution (Sigma-Aldrich) and monitored for clear area (band) formation. Images were captured using the Bio-Rad ChemiDoc imaging system.

For the quantitative measurement of *in vitro* ferroxidase activity, equivalent amounts of lysates were incubated with 30 μL of anti-DDK Beads (Origene) for 4 h at 4^o^ C with rotation. After incubation, beads were washed three times with PBS containing 0.5% Triton X100 and re-suspended in 0.5 mg/mL ferrous ammonium sulfate solution prepared in 0.1 M sodium acetate, pH 5.0, and samples were then incubated at 37^o^ C for 1.5 h (50 μL total reaction volume). Following incubation, 250 μL of 0.1 M sodium acetate and 30 μL non-reducing iron detection reagent (6.5 mM Ferrozine, 2.5 M ammonium acetate) were added to the beads, and beads were further incubated at RT for 30 min. After centrifugation of samples for 1 minute at 10,000 rpm, 280 μL of each sample was carefully transferred to a 96 well plate, and the iron concentration was measured by absorbance at 550 nm using a SpectraMax plate reader. Purified human ceruloplasmin (25 μL; Athens Research and Technology), used as positive control, was diluted in 0.1M sodium acetate pH 5.0 and incubated with 25 μL of 0.5 mg/mL ferrous ammonium sulfate and processed identically in parallel. Iron standards were prepared by adding 0, 5, 10, and 25 μL of 1 mM iron standard (Bio Vision) to a final volume of 50 μL in 0.1 M sodium acetate, pH 5.0, and processed identically as described above, except for the use of a reducing iron detection reagent [6.5 mM ferrozine, 2.5 M ammonium acetate, 1 M ascorbic acid] in place of the non-reducing detection reagent. Ceruloplasmin was used to show that the *in vitro* ferroxidase assay can detect the conversion of Fe (II) to Fe (III) in an enzyme concentration-dependent manner.

### Mass spectrometry

HEK293 cells were transiently transfected with WT-HEPHL1, HEPHL1 M1059T, or HEPHL1 Δexon 5 constructs. After transfection, cells were collected, washed with PBS and suspended in 62.5 mM Tris-HCl (pH 6.8), and then an equal amount of 2X SDS buffer (62.5 mM Tris-HCl [pH 6.8], 6% SDS, 10% glycerol, 20 mM iodoacetamide, protease and phosphatase inhibitors) was added. Cell lysates were sonicated and centrifuged and diluted ten times with Triton X-100 buffer (50 mM Tris-HCl [pH 7.5], 150 mM NaCl, 0.5% Triton X-100, 1 mM EDTA, 10 mM iodoacetamide, protease and phosphatase inhibitors). Equivalent amounts of lysates were incubated with anti-DDK agarose beads (Origene; clone OT14C5) for 4 h at 4°C with rotation. After incubation, immunoprecipitates were washed three times with Triton X-100 buffer. For the glycosylation analysis, enzymatic release of glycans from the immunoprecipitated HEPHL1 was performed directly on beads using NEB deglycosylation mix II to remove potential *N*-linked and/or *O*-linked glycans. The deglycosylated or non-deglycosylated protein bands were excised from the gel and digested using chymotrypsin and trypsin followed by the Liquid Chromatography-tandem Mass Spectrometry (LC-MS/MS) analysis (see S2 Text for a full description of the method and instrument used for LC-MS/MS).

### Measurement of intracellular iron

Quantitation of intracellular iron concentration in dermal fibroblasts was performed as described by Reimer et al. [44]. Briefly, cells were lysed using 50 mM NaOH for 2 h, neutralized by addition of equal volume of 10 mM HCl and sonicated. Protein concentration was determined using the DC protein assay, and lysates were then treated with freshly prepared acidic KMnO_4_ solution (1.4M HCl and 4.5% (w/v) KMnO_4_ in H_2_O) at 60°C for 2 h with shaking. The total iron content of the lysates was determined by the addition of an iron detection reagent (6.5 mM ferrozine, 1 M ascorbic acid, 2.5 M ammonium acetate) followed by measurement of absorbance at 550 nm. To measure the expression of ferritin in fibroblasts, a rabbit polyclonal anti-ferritin heavy chain antibody (Santa-Cruz Biotechnology, sc-25617) was used.

### Measurement of lysyl oxidase

Lysyl oxidase activity was measured using an Amplite Fluorimetric Lysyl Oxidase Assay Kit (AAT Bioquest Inc., Sunnyvale, CA) following the manufacturer’s instructions. Briefly, 50 μL of cell culture medium from fibroblasts growing in the presence or absence of the lysyl oxidase inhibitor 2-aminopropionitrile (BAPN, Sigma-Aldrich) were collected and mixed with an equal volume of assay mixture containing horseradish peroxidase (HRP) and Amplite HRP substrate. After incubation at 37°C, the change in fluorescence was measured using a fluorescence plate reader (Molecular Probes) at Ex/Em 540/590 nm. The specific fluorescence attributed to lysyl oxidase activity in each sample was determined after subtracting the fluorescence obtained in the presence of BAPN. Total protein contents were determined after lysis of cells in SDS buffer and used for the normalization of lysyl oxidase activity. To visualize different forms of lysyl oxidase, unaffected controls and patient’s fibroblasts were grown to confluence in T-75 flasks and then incubated with phenol red and serum-free medium for another two days. Equal amounts of conditioned cell media were collected and concentrated using an ultra-15 centrifugal filter unit (EMD Millipore). Cell lysates were prepared by lysis of cells in Triton X-100 buffer (150 mM NaCl, 1% Triton X-100 and 50 mM Tris (pH 8.0), with protease and phosphatase inhibitors). Western blot analysis was performed using a LOX specific antibody (Abcam, Ab174316).

### Generation of *Hephl1* knockout mice

Heterozygous mice with conditional (floxed) *Hephl1* allele were commercially created for us by the UC Davis Mouse Biology Program. The targeting construct used to generate the mice contained LoxP sites that flanked exon 2, a 11.5 kb 5’ arm of homology (containing exon 1), a 11.2 kb 3’ arm of homology (containing exons 3–6 and the 5’ portion of exon 7), a Diphtheria Toxin A (DTA) cassette, and a Neomycin (neo) cassette flanked by FRT sites. JM8.F6 C57BL/6N embryonic stem (ES) cells were microinjected with the targeting construct and one colony that successfully incorporated the targeting construct and had a high percentage of cells (88%) with the expected karyotype was selected. The cells were injected into BALB/c blastocysts and implanted into pseudo-pregnant females. Three high percentage chimeric males were generated (estimated 75–85% C57BL/6 cells based on coat color) and bred with wild-type C57BL/6 females. Black pups were genotyped and females heterozygous for the *Hephl1* floxed allele (still bearing the FRT-flanked neo cassette (*Zp*^*fl(neo)/+*^) were used for further breeding. In order to remove the neo cassette, the *Zp*^*fl(neo)/+*^ females were crossed with a C57BL/6J mouse bearing the FlpE transgene [(“FlpE” mouse, B6. Cg- Tg(ACTFLPe)9205Dym/J, The Jackson Laboratory) kindly provided by Dr. Ian Tonks at QIMR Berghofer]. When pups inherit a copy of the FlpE transgene, the FlpE recombinase protein is expressed and excises regions of DNA flanked by FRT sites, in this case the neo cassette.

To make the *Hephl1* floxed strain (Hephl1^tm1.1Vul^, denoted as *Zp*^*fl/fl*^ hereafter), the *Zp*^*fl/+*^ FlpE mice lacking the neo cassette were first bred with wild-type C57BL/6J mice, and then those containing a *Hephl1* floxed allele, but lacking the FlpE transgene (Zp^fl/+^), were backcrossed onto the C57BL/6J strain for multiple generations until at least 97% C57BL/6J. These *Zp*^*fl/+*^ mice were then bred together to generate *Zp*^*fl/fl*^ mice. To make the whole body *Hephl1* knockout strain (Hephl1^tm1.2Vul^, hereafter referred to as *Zp*^*-/-*^), *Zp*^*fl/fl*^ mice were bred with C57BL/6J mice bearing the Cre recombinase transgene driven by the eIIa promoter, which is ubiquitously active (“eIIa-cre” mice, B6.FVB-Tg(EIIa-cre) C5379Lmgd, The Jackson Laboratory). Expression of Cre recombinase leads to excision of the region in the DNA between the LoxP sites (exon 2 of *Hephl1*). Progeny containing both the eIIa-cre transgene and the *Hephl1* floxed allele (Zp^fl/+^ EIIa) were then bred together and pups that likely contained germline deletion of *Hephl1* were selected by genotyping. These mice were then backcrossed onto the C57BL/6J line to remove both the FlpE and eIIa-cre recombinase transgenes and to generate mice at least 97% C57BL/6J and heterozygous for the *Hephl1* knockout allele (*Zp*^*+/-*^). To generate *Zp*^*-/-*^ for this study, *Zp*^*+/-*^ mice were then bred together. Genotyping was done by PCR using forward primer CCTTATGACTACAGTGAACAGGGTTCTG and reverse primer CTACTCTCTGGCCCTTGCTTTTGC to amplify the wild-type allele and forward primer CGACGGCCAGTGAATTGTAATA and reverse primer GTGATAGAGCTGAGATGGCGCAA to amplify the knockout allele.

### Analysis of *Hephl1* mRNA expression in mouse tissues

Quantitative analysis of *Hephl1* mRNA expression was carried out using total RNA extracted from *Zp*^+/+^ and *Zp*^-/-^ frozen placental tissues by TRIzol reagent (Thermo Fisher Scientific). cDNA was synthesized using M-MLV Reverse Transcriptase (Thermo Fisher Scientific) and an oligo (dT) primer as per manufacturer’s instructions. Real-time quantitative PCR was performed with a CFX 384 detection system (Bio-Rad) using iTaq Universal SYBR Green supermix. Each sample was analyzed in triplicate and gene expression was calculated from the Cq value using the standard curve method. Gene expression levels were normalized to the expression of the housekeeping gene hypoxanthine guanine phosphoribosyltransferase (*Hprt*). Primer validation and analyses were in accordance with the MIQE guidelines[45]. The primer pairs used were: *Hephl1* Exon 2 forward: GGTGGGATCTACAAGAAGGCG; *Hephl1* Exon 2 reverse: GTCTCCCACTTCTGCCCTCA; *Hephl1* Exon 18–19 forward: GTTTGCTGATCACCCAGGAACA; *Hephl1* Exon 18–19 reverse: TCCAGAAGGCGTCTTGGTAGAAT; *Hprt* forward: GGACTGATTATGGACAGGA; *Hprt* reverse: GAGGGCCACAATGTGATG.

### Measurement of lysyl oxidase activity in mouse embryonic fibroblasts

Mouse embryonic fibroblasts (MEFs) were derived from embryonic day (E)12.5 embryos according to established procedures [46, 47] and cultured using high glucose DMEM (Thermo Fisher Scientific) supplemented with 10% FBS, 1% GlutaMAX (Thermo Fisher Scientific) and 1% penicillin–streptomycin. For the measurement of lysyl oxidase activity, MEFs of defined genotype at passage 3–4 were seeded into 12-well plates at a density of 2.5 x10^5^ cells/well. When the cells reached confluence, the medium was carefully aspirated and replaced with fresh phenol red-free complete culture medium. Twenty-four hours later, 500 μM of BAPN was added to the wells. After another 24 hours, the cell surface was flushed gently several times with the existing culture medium, which was then collected for lysyl oxidase (LOX) activity measurement. LOX activity was measured as described for human fibroblasts. Cells were then washed once with PBS, and 200 μL of lysis buffer (25 mM Tris-HCl, pH 7.2, 25 mM NaCl with 2% SDS) was added into each well to lyse the cells. Total protein contents from the clarified supernatants of the cell lysates were determined by BCA protein assay (Thermo Scientific) and used for normalization of the fluorescence readings. The percentage of lysyl oxidase-specific fluorescence for each MEF cell line was calculated by dividing the difference in fluorescence between untreated and BAPN-treated wells by the fluorescence obtained from the untreated wells.

## Supporting information

S1 Fig *HEPHL1* mRNA expression in fibroblasts and iPS cells. Quantitative real time PCR analysis showed several folds higher *HEPHL1* mRNA expression in iPS cells compared to fibroblasts. Values are mean ± SD, n = 3.
(TIF)

S2 Fig Measurement of ferroxidase activity of ceruloplasmin (CP). Ferroxidase activity of CP is indicated by reduction in the amount of Fe (II) iron. Ferrozine binds Fe (II), but not Fe (III), and forms a complex that absorb at 550 nm. The ferroxidase activity of CP converts Fe (II) to Fe (III) leading to reduction in absorbance. Values are mean ±SD, n = 5.
(TIF)

S3 Fig HEPHL1 peptides identified by LC-MS/MS. Illustration of peptide areas detected by LC-MS/MS analyses of WT-HEPHL1 (upper), M1059T mutant (middle) and Δexon 5 mutant (lower) samples. Peptides detected with high confidence (1% false discovery rate) are highlighted in green. *N*-linked deglycosylated peptides and glycosylation on asparagine sites were identified by comparing deglycosylation enzyme mix treated samples with the untreated. The peptides containing a site of *N*-linked glycosylation were determined by the identification of peptides with a conversion of Asn to Asp (a molecular weight addition of 0.984 Da), caused by removal of the entire carbohydrate from the side chain of asparagines using deglycosylation enzyme mix. Without deglycosylation (the untreated sample), the glycan-free peptide was not present and therefore could not be detected by LC-MS/MS. There are five conserved *N*-linked glycosylation motifs (NXT/S) in human HEPHL1 (underlined). Three sites (N^161^YT, N^407^AS and N^772^RT) were identified in WT-HEPHL1 (bold and underlined). Two sites (N^161^YT and N^772^RT) were identified in M1059T mutant (bold and underlined) while no *N*-linked glycosylation site was identified in the Δexon 5 mutant. Sites of *O*-linked glycosylation were determined by identification of peptides with the HexNAc (S/T O-GlcNAc) modification on serine/threonine (+203.079 Da). *O*-linked glycosylation was detected on S1076 and T1077 in WT and the M1059T mutant, and S991 and T992 in the Δexon 5 mutant (shown in red).
(TIF)

S1 Text Clinical report of the patient. (DOCX)

S2 Text Method and instrument used for LC-MS/MS analysis. (DOCX)
