# Supplementary figures and images for: Biallelic HEPHL1 variants impair ferroxidase activity and cause an abnormal hair phenotype
Source: PLoS Genet. 2019 May 24;15(5):e1008143. doi: 10.1371/journal.pgen.1008143 (PMC6534290; doi:10.1371/journal.pgen.1008143)

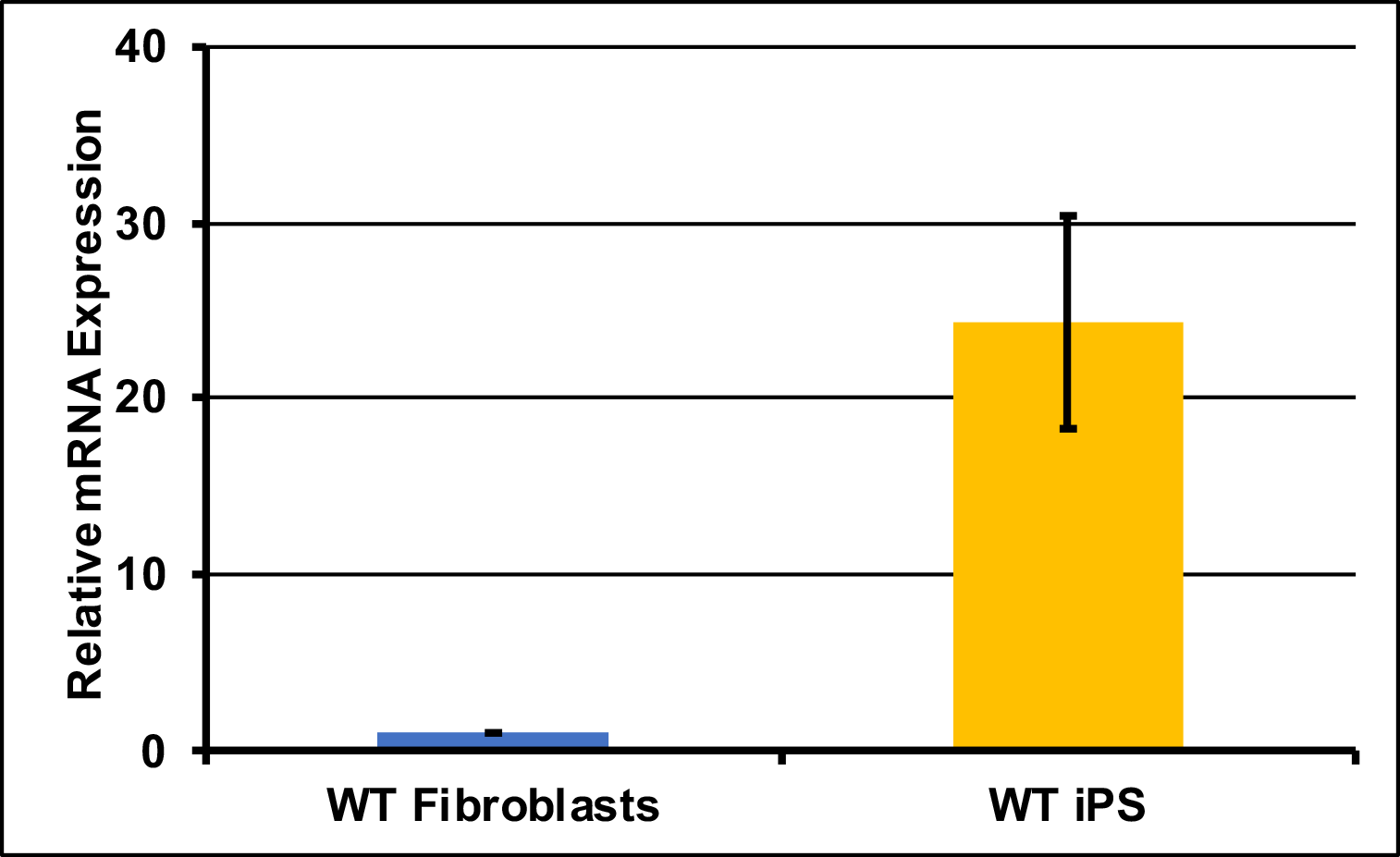

Supplement: S1 Fig — Quantitative real time PCR analysis showed several folds higher HEPHL1 mRNA expression in iPS cells compared to fibroblasts. Values are mean ± SD, n = 3. (TIF) [file pgen.1008143.s001.tif]

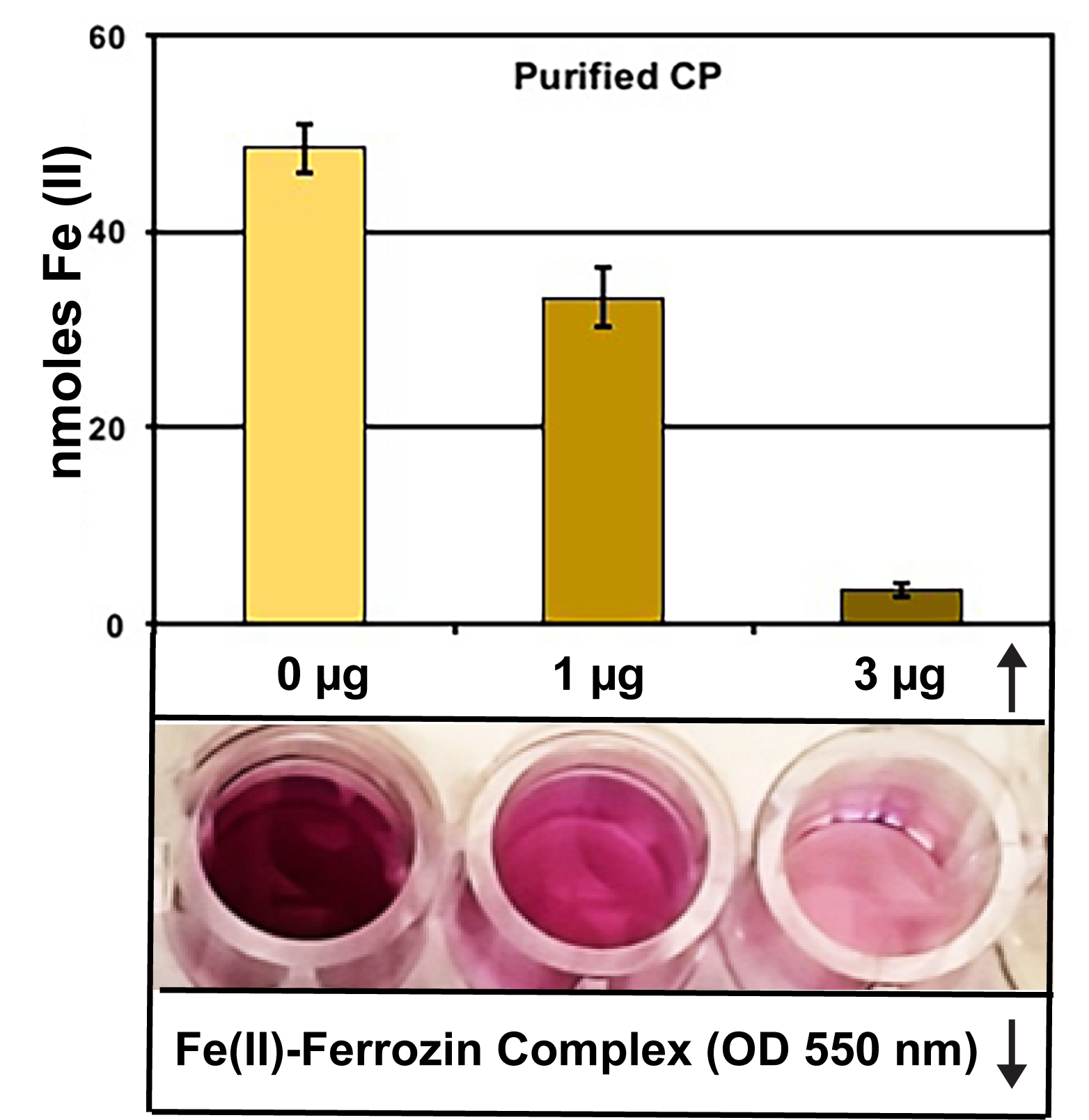

Supplement: S2 Fig — Ferroxidase activity of CP is indicated by reduction in the amount of Fe (II) iron. Ferrozine binds Fe (II), but not Fe (III), and forms a complex that absorb at 550 nm. The ferroxidase activity of CP converts Fe (II) to Fe (III) leading to reduction in absorbance. Values are mean ±SD, n = 5. (TIF) [file pgen.1008143.s002.tif]

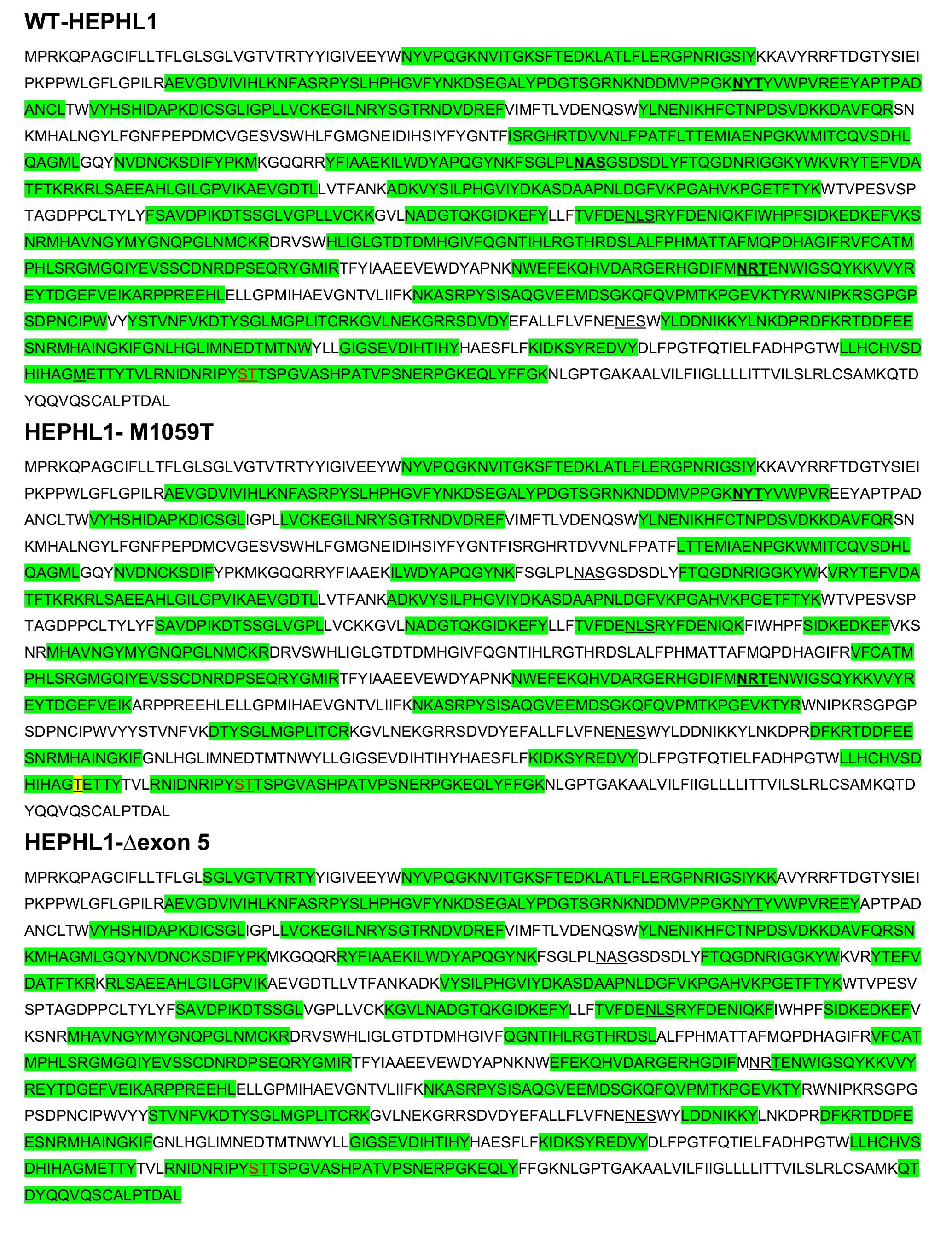

Supplement: S3 Fig — Illustration of peptide areas detected by LC-MS/MS analyses of WT-HEPHL1 (upper), M1059T mutant (middle) and Δexon 5 mutant (lower) samples. Peptides detected with high confidence (1% false discovery rate) are highlighted in green. N-linked deglycosylated peptides and glycosylation on asparagine sites were identified by comparing deglycosylation enzyme mix treated samples with the untreated. The peptides containing a site of N-linked glycosylation were determined by the identification of peptides with a conversion of Asn to Asp (a molecular weight addition of 0.984 Da), caused by removal of the entire carbohydrate from the side chain of asparagines using deglycosylation enzyme mix. Without deglycosylation (the untreated sample), the glycan-free peptide was not present and therefore could not be detected by LC-MS/MS. There are five conserved N-linked glycosylation motifs (NXT/S) in human HEPHL1 (underlined). Three sites (N161YT, N407AS and N772RT) were identified in WT-HEPHL1 (bold and underlined). Two sites (N161YT and N772RT) were identified in M1059T mutant (bold and underlined) while no N-linked glycosylation site was identified in the Δexon 5 mutant. Sites of O-linked glycosylation were determined by identification of peptides with the HexNAc (S/T O-GlcNAc) modification on serine/threonine (+203.079 Da). O-linked glycosylation was detected on S1076 and T1077 in WT and the M1059T mutant, and S991 and T992 in the Δexon 5 mutant (shown in red). (TIF) [file pgen.1008143.s003.tif]
