# Supplementary material for: Biallelic HEPHL1 variants impair ferroxidase activity and cause an abnormal hair phenotype
Source: PLoS Genet. 2019 May 24;15(5):e1008143. doi: 10.1371/journal.pgen.1008143 (PMC6534290; doi:10.1371/journal.pgen.1008143)
Supplement: S1 Text — (DOCX) [file pgen.1008143.s004.docx]

**Clinical characteristics**

The proband is a twelve-year old Caucasian male of non-consanguineous Native American and Mexican descent, born at 38-weeks gestation following the spontaneous rupture of membranes and complicated by the breech position, requiring cesarean section. Fetal movements, ultrasounds, and laboratory screens were all normal. Birth weight was 2.13 kg, length 47 cm. At birth, his hair was full, rich, and black and had a normal, even distribution. He did not have eyebrows but did have full lashes. He lost weight prior to frenulectomy and transition to formula at one-week of age. He experienced frequent vomiting up to three months of age and required transition to a moderately better tolerated soy-based diet. Head CT at 5 months showed left parieto-occipital plagiocephaly, without craniosynostosis. The infant experienced anterior hair loss that progressed to total alopecia by six-months of age. He had eight episodes of otitis media between 6 and 12 months of age. A formal hearing evaluation did not identify any deficit and tympanostomy tubes were not placed. He developed normally in the first year of life, meeting all milestones except in speech.

The boy was formally evaluated at three-years of age when his mother noticed that he experienced a regression in acquired skills for counting and identifying known colors. CAT/CLAMS testing of visual-motor and problem-solving skills indicated a developmental age of 36 months except for language skills, which were equivalent to age 21 months with poor articulation. The child experienced episodes of fatigue lasting several days, commonly precipitated by hot environments. Fevers required IV fluid management. He had frequent leg pain from the ankle to knee that awakened him from sleep at night. He experienced nasal allergies without epistaxis, or sinusitis, and had a history of snoring since infancy. The proband demonstrated significant behavior problems and impulsivity, with aggression toward other children. After an extensive neuro-psych evaluation he was diagnosed with ADHD. He has markedly improved age-appropriate behavior on guanfacine and fluoxetine with readily-understandable language and cognitive delays managed with speech therapy. Family history included learning disabilities, and substance abuse on both the maternal and paternal sides. Maternal family history was significant for dysmorphology (anophthalmia, bilateral cleft lip and palate, finger-toe abnormalities). Medications included guanfacine, fluoxetine, and polyethylene glycol. The boy received all recommended childhood vaccines with known hypersensitivity only to trimethoprim-sulfamethoxazole.

Evaluation at the National Institutes of Health Clinical Center identified a well-nourished, appropriately developed preschooler, in the 25-50^th^ percentile for weight and 50-75^th^ percentile for height. He exhibited elfin facies, missing the lateral third of his eyelashes, and had sparse eyebrows. He had increased skin markings of the superior eyelids and infraorbital Dennie-Morgan folds. The palpebral fissure slants were neutral and symmetrical. Inner canthal distance and inter-pupillary distance were neutral. Irides were light blue without ptosis, nystagmus, or coloboma but slight left esotropia. Funduscopic examination demonstrated photosensitivity but no visual defects. He had mild occipital flattening and hair that grew in a patchy distribution, with more along the crown and sparser distribution in the temporal areas. Samples showed pili torti and trichorrhexis nodosa on light microscopy. He exhibited mild nasal congestion and a high-arched palate. His nasal bridge, nasal tip, and philtrum length were normal. The maxilla and mandible were normal but formed a slightly small mouth. His tongue laid midline. Teeth appeared small and slightly discolored, with a small space between the upper central incisors. Ears were normally positioned without pits or tags. Tympanic membranes were normal. He passed repeated hearing tests despite recurrent otitis media in infancy. His skin was smooth and non-ichthyotic without evidence of follicular atrophoderma. He had extensive numbers of adnexal hamartomas, along with midline chest and axillary vellus hair cysts that were erupting. He had a 2-cm café-au-lait lesion on his left clavicle. Keratosis pilaris eruptions on the legs were concentrated particularly on the knees, anterior shins, and upper thighs. Palms and soles were not hyperkeratotic but he had brittle, hypoplastic nails, loose joints and short fourth, fifth metatarsals. Fingers and toes demonstrated normal dermatoglyphics and numbers of functional sweat glands. He did not have a pectus, and the inter-nipple distance was normal. Auscultation confirmed clear S1-S2 in normal sinus rhythm. Femoral pulses were strong and present bilaterally.

Electrocardiogram revealed sinus bradycardia with multiple atrial premature complexes, SV complexes, and short RR intervals. Echocardiogram identified normal left and right ventricle size and function without regional wall motion abnormalities. There was no evidence of atrial/ventricular septal defect, patent ductus, coarctation or pulmonary hypertension. There was, however, mild mitral, tricuspid, and pulmonic regurgitation. There was no evidence of aortic regurgitation. He demonstrated slightly congested cough but his chest was clear to auscultation bilaterally. His abdomen had normal bowel sounds in all quadrants and was soft and non-tender without hepatosplenomegaly. He had normal male Tanner stage I genitalia with testes descended bilaterally, normal anus, without sacral dimpling. Rectal vault was dilated on exam. Fecal occult blood tests were negative. His left shoulder hung lower than his right. Joint popping was frequently appreciated, particularly at the neck, shoulders and arms. He had no dislocations but mobility was increased in all joints, particularly the thumbs. He had increased carrying angle at the elbows. He had no erythema, swelling or bruising associated with his leg pain, but his toes showed mild 4-5 clinodactyly and 2-3 syndactyly. He was unable to respond to directions until administration of guanfacine, after which his impulsive behavior and hyperactivity quieted. Cranial nerves II-XII were grossly intact. He had a normal, well-balanced gait and could walk on toes and heels. Muscle tone, strength, and tendon reflexes were normal.

Cytogenetics by Quest Diagnostics revealed a normal, 46XY karyotype. No mutations associated with Ectodactyly-ED-Clefting, Hey-Wells, or related syndromes were detected. Routine labs revealed a normal CMP, Chem20, ESR, iron studies, lipid profiles, TSH, free T4, 1, 25-dihydroxy-vitamin D, plasma amino acids. Carnitine and acylcarnitine profiles were normal. Copper, ceruloplasmin, ammonia levels, and quantitative immunoglobulins including IgE were normal. Head CT and audiograms revealed no abnormalities. Plasma catecholamines included norepinephrine (95 pg/mL; normal 80-498), epinephrine (estimation <23 pg/ml interfering peak present), dopamine (17 pg/mL; normal 3-46). CSF catecholamine studies were not performed nor were nerve conduction studies or amino acid analyses. A protein-load plasma amino acid profile was performed with results of slightly increased methionine 50 (normal 7-47 µmol/L), slightly increased tyrosine 127 (normal 24-115 µmol/L), slightly increased lysine 335 (normal 48-284 µmol/L). Urinalysis was normal. Myoglobin and organic acid profiles were normal. Abdominal x-ray images demonstrated a nonspecific bowel gas and feces pattern with moderate right and left colon feces and no evidence of obstruction. Abdominal ultrasound showed no evidence of perisplenic, perihepatic or lesser sac fluid collection, intrahepatic biliary dilatation or liver mass. Pancreas, gallbladder, and main portal flow appeared normal. Kidney lengths were at the lower limits of normal for age and at or below the lower limit of normal for body weight. Spleen length was within normal limits for age and weight. Skeletal survey demonstrated normal skull and cervical-thoracic spine. Lumbar spine exhibited increased interpedicular distances on the frontal view. Upper extremities and hands were normal. Lower extremities demonstrated flaring at the metadiaphysis of the distal femurs with small growth arrest lines in the distal tibias. Feet and ankles were normal. Bone age was appropriate for chronological age according to the Greulich and Pyle standard.

Whole exome sequencing of DNA from patient and unaffected parents identified variants in several genes. Among these genes, variants identified in *HEPHL1*, *ZIM2 and ZNF157* were prioritized based on coding effect (nonsynonymous, frameshift, stopgain, stoploss, startloss), proximity to splice sites (within 20 base pairs of a canonical splice site into the intron, or 5 base pairs into the exon), and CADD Phred scores. Subsequent analysis of *ZIM2* variant (NM_001146326.1; c.1072T>C; pCys358Arg) reveled higher frequency (0.01114%) and presence of 6 homozygotes in ExAc population frequency data base (see URL). *ZNF157* variant (NM_003446.3; c.1366C>T; pArg456Cys) was also ruled out based on low CADD Phred score (14.94) and predicted as benign by Polyphen and Sift.
