# Supplementary material for: Biallelic HEPHL1 variants impair ferroxidase activity and cause an abnormal hair phenotype
Source: PLoS Genet. 2019 May 24;15(5):e1008143. doi: 10.1371/journal.pgen.1008143 (PMC6534290; doi:10.1371/journal.pgen.1008143)
Supplement: S2 Text — (DOCX) [file pgen.1008143.s005.docx]

## Mass spec analysis

Peptide samples were analyzed by LC-MS/MS. LC-MS/MS analyses were performed using a Thermo Scientific Q-Exactive hybrid Quadrupole-Orbitrap Mass Spectrometer and a Thermo Dionex UltiMate 3000 RSLCnano System. For each LC-MS/MS run the peptide mixture was loaded onto a peptide trap cartridge at a flow rate of 5μL/min. The trapped peptides were eluted onto a reversed-phase PicoFrit column (New Objective, Woburn, MA) using a linear gradient of acetonitrile (3-36%) in 0.1% formic acid. The elution duration was 110 min at a flow rate of 0.3μL/min. Eluted peptides from the PicoFrit column were ionized and sprayed into the mass spectrometer, using a Nanospray Flex Ion Source ES071 (Thermo) under the following settings: spray voltage, 1.6 kV, Capillary temperature, 250°C. Other settings were empirically determined. For protein identification, two raw MS files from two LC-MS/MS runs for each sample were analyzed using the Thermo Proteome Discoverer 1.4.1 platform (Thermo Scientific, Bremen, Germany) for peptide identification and protein assembly. The human protein database obtained from the NCBI website was searched based on the SEQUEST and percolator algorithms through the Proteome Discoverer 1.4.1 platform; in addition, the database was manually modified on the human HEPHL1 mutations. Carbamidomethylation (+57.021 Da) of cysteines was set as a fixed modification; oxidation, deamidation Q/N-deamidated (+0.98402 Da) and S/T O-GlcNAc (+203.0788Da) were set as dynamic modifications. The minimum peptide length was specified to be five amino acids. The precursor mass tolerance was set to 15 ppm, whereas fragment mass tolerance was set to 0.05 Da. The maximum false peptide discovery rate was specified as 0.01. The resulting Proteome Discoverer Report contains all assembled proteins with peptide sequences and matched spectrum counts. The estimation of relative abundance of protein is based on peptide spectrum match counts (PSM).
